# Structural Genomic Variation as Risk Factor for Idiopathic Recurrent Miscarriage

**DOI:** 10.1002/humu.22589

**Published:** 2014-06-24

**Authors:** Liina Nagirnaja, Priit Palta, Laura Kasak, Kristiina Rull, Ole B Christiansen, Henriette S Nielsen, Rudi Steffensen, Tõnu Esko, Maido Remm, Maris Laan

**Affiliations:** 1 Human Molecular Genetics Research Group, Institute of Molecular and Cell Biology, University of TartuTartu, Estonia; 2 Department of Bioinformatics, Institute of Molecular and Cell Biology, University of TartuTartu, Estonia; 3 Institute for Molecular Medicine Finland (FIMM), University of HelsinkiHelsinki, Finland; 4 Wellcome Trust Sanger Institute, Wellcome Trust Genome CampusCambridge, United Kingdom; 5 Department of Obstetrics and Gynaecology, University of TartuTartu, Estonia; 6 The Fertility Clinics, Rigshospitalet, Copenhagen University HospitalCopenhagen, Denmark; 7 Department of Obstetrics and Gynaecology, Aalborg University HospitalAalborg, Denmark; 8 Department of Clinical Immunology, Aalborg University HospitalAalborg, Denmark; 9 Estonian Genome Center, University of TartuTartu, Estonia; 10 Children's Hospital BostonBoston, Massachusetts; 11 Medical and Population Genetics, Broad InstituteCambridge, Massachusetts; 12 Estonian BiocentreTartu, Estonia

**Keywords:** recurrent miscarriage, fetomaternal interface, immune dysfunction, placenta, *GOLPH3*, *PDZD2*

## Abstract

Recurrent miscarriage (RM) is a multifactorial disorder with acknowledged genetic heritability that affects ∼3% of couples aiming at childbirth. As copy number variants (CNVs) have been shown to contribute to reproductive disease susceptibility, we aimed to describe genome-wide profile of CNVs and identify common rearrangements modulating risk to RM. Genome-wide screening of Estonian RM patients and fertile controls identified excessive cumulative burden of CNVs (5.4 and 6.1 Mb per genome) in two RM cases possibly increasing their individual disease risk. Functional profiling of all rearranged genes within RM study group revealed significant enrichment of loci related to innate immunity and immunoregulatory pathways essential for immune tolerance at fetomaternal interface. As a major finding, we report a multicopy duplication (61.6 kb) at 5p13.3 conferring increased maternal risk to RM in Estonia and Denmark (meta-analysis, *n* = 309/205, odds ratio = 4.82, *P* = 0.012). Comparison to Estonian population-based cohort (total, *n* = 1000) confirmed the risk for Estonian female cases (*P* = 7.9 × 10^−4^). Datasets of four cohorts from the Database of Genomic Variants (total, *n* = 5,846 subjects) exhibited similar low duplication prevalence worldwide (0.7%–1.2%) compared to RM cases of this study (6.6%–7.5%). The CNV disrupts *PDZD2* and *GOLPH3* genes predominantly expressed in placenta and it may represent a novel risk factor for pregnancy complications.

## Introduction

Miscarriage is the most common pregnancy complication affecting up to 15% of all clinically recognized pregnancies [Stirrat, 1990]. The risk of miscarriage increases with age and is enhanced by a trend in developed countries to postpone childbearing to late 30s and early 40s with declined fertility rates [Nybo Andersen et al., 2000; Group TECW, 2010]. Recurrent miscarriage (RM), defined as ≥3 consecutive pregnancy losses before 22nd gestational week, is a heterogeneous disorder affecting up to 3% of couples aiming at childbirth [Christiansen et al., 2008]. It is a distressing condition for affected couples as each subsequent miscarriage leads to elevated risk of experiencing further pregnancy loss [Ogasawara et al., 2000] and increased probability of other pregnancy complications such as preterm delivery or small for gestational age newborns [Jivraj et al., 2001; van Oppenraaij et al., 2009]. Although a spectrum of factors is known to increase the risk to RM, including immune system dysfunction and thrombophilic disorders, the underlying etiology remains undetermined in about half of the cases [Rai and Regan, 2006; Allison and Schust, 2009].

A familial segregation of RM has been observed highlighting the contribution of the genetic predisposition to the disease [Christiansen et al., 1990; Kolte et al., 2011]. So far, majority of the studies focusing on the genetic susceptibility to RM have addressed single nucleotide polymorphisms (SNP) in more than 100 RM candidate genes, often providing conflicting results and no identified risk variants with confirmed strong effect [Nagirnaja et al., 2012; Rull et al., 2012, 2013]. More recently, paternal DNA fragmentation, epigenetic disturbances, and DNA copy number variants (CNVs) have been suggested to modify predisposition to RM [Rajcan-Separovic et al., 2010; Uuskula et al., 2010; Robinson et al., 2012]. CNVs represent deletions or duplications of >50 bp DNA sequence that disrupt around 13% of RefSeq genes [McCarroll et al., 2008; Zhang et al., 2009; Conrad et al., 2010]. It has been estimated that 17.7% of variability in gene expression may be attributed to these DNA variants and, subsequently, a growing number of genic CNVs and increased global burden of CNVs have been linked to several complex disorders [Stranger et al., 2007; Zhang et al., 2009; Girirajan et al., 2011]. Studies focusing on structural variation predisposing to pregnancy complications have been scarce and represent single reports addressing disease-associated CNVs in unexplained stillbirths and preeclampsia [Harris et al., 2011; Zhao et al., 2012, 2013]. A single study has addressed the contribution of CNVs in RM using array comparative genomic hybridization to screen 26 placentas from RM pregnancies for rare CNVs to identify candidate genes, which could be causative for the specific miscarriage [Rajcan-Separovic et al., 2010].

**Figure 1:**
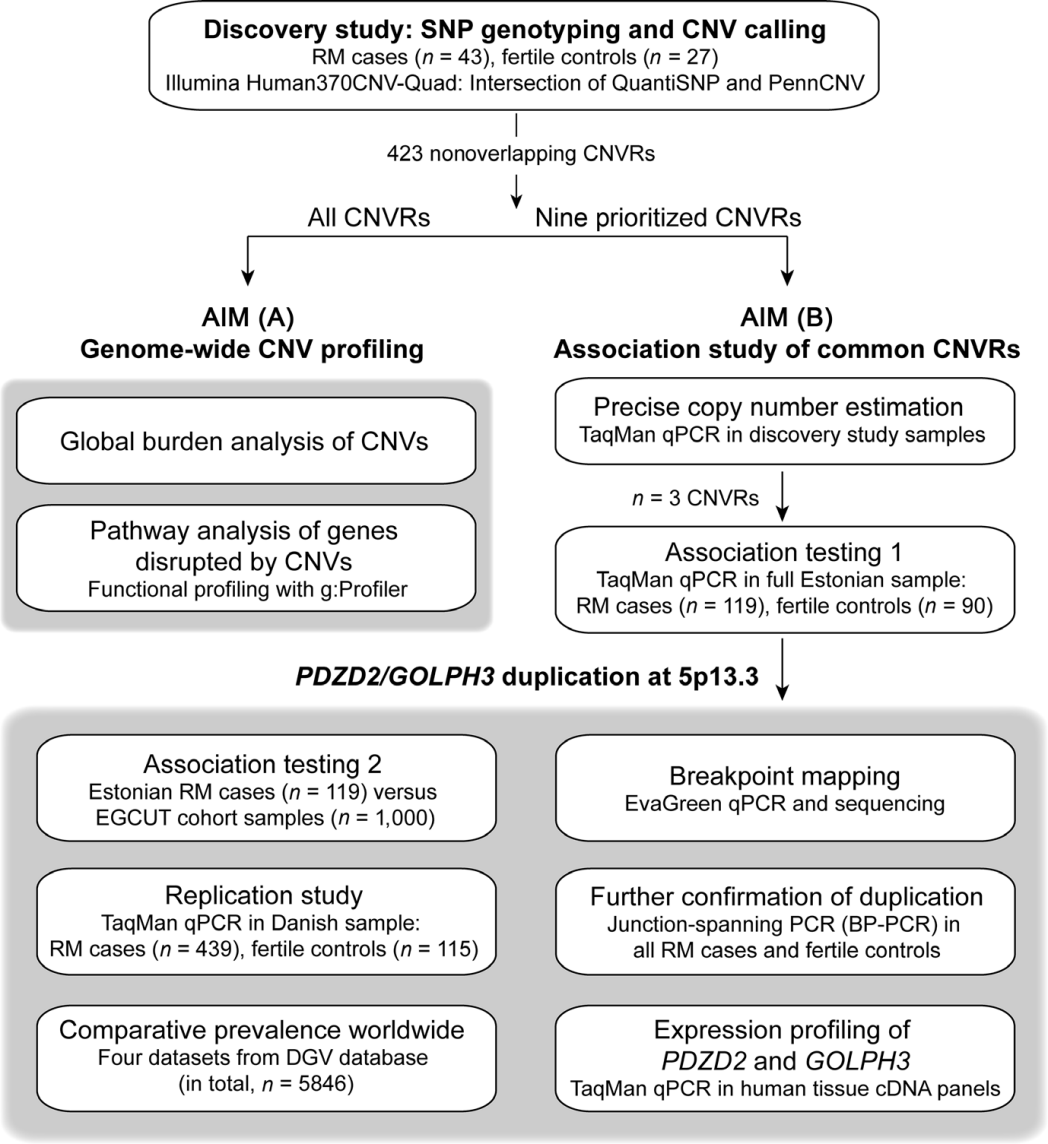
Study design and procedures. Following initial CNV discovery phase in a subsample of Estonian subjects, whole-genome profiling of all identified CNVs and association study of discrete CNV regions were performed. Three CNVRs were taken forward to be analyzed in the full Estonian sample set to identify potential risk-conferring rearrangements. For the duplication at 5p13.3, genetic Association testing 1 in the full Estonian sample and Association testing 2 using Estonian cohort (EGCUT) control samples were then undertaken, followed by Replication study in the Danish sample set. Comparative prevalence data from the Database of Genomic Variants (DGV) were collected followed by detailed duplication breakpoint characterization and confirmation of duplication carriership of all RM cases and fertile controls of the study using duplication-specific junction-spanning PCR. Expression profiling in human tissues was performed for the genes *PDZD2* and *GOLPH3* disrupted by the 5p13.3 rearrangement.

In the current genome-wide study, we set forward to address the role of CNVs in predisposing to RM among the couples of unexplained RM from Estonia and Denmark. The collaborative effort involved 558 RM cases with ≥3 consecutive miscarriages (in total, 309 RM female and 249 male partners), fertile multiparous control women (*n* = 205), and population-based samples from Estonian Biobank, Estonian Genome Center, University of Tartu (EGCUT; *n* = 1,000). We aimed to (A) assess the contribution of genome-wide burden of CNVs and (B) identify and characterize novel common CNVs in modulating the risk of RM (Fig.1). We determined excessive cumulative burden of all CNVs (5.4 and 6.1 Mb per genome) in two RM cases possibly increasing their individual risk of the disease. The genomic CNV profile within the RM study group was significantly enriched for CNVs predominantly affecting genes related to innate immunity signaling and immunoregulatory interactions. As a major finding, we identified a multicopy duplication CNV (61.6 kb) at 5p13.3 conferring high, almost fivefold increased maternal risk of RM and disrupting two novel RM candidate genes, *PDZD2* and *GOLPH3*, associated with pregnancy maintenance for the first time.

## Materials and Methods

### Study Subjects

#### RM cases and fertile control samples

RM cases and fertile controls have been recruited in two North European countries Estonia and Denmark, and the applied sample sets have been well characterized and previously exploited in RM research [Kruse et al., 2004; Rull et al., 2008, 2013; Kolte et al., 2011]. The study was approved by the Ethics Review Committee on Human Research of the University of Tartu, Estonia, and the Danish Central National Committee on Biomedical Research Ethics.

As both maternal and paternal genetic information determine the development of the fetus and the placenta, as well as the outcome of a pregnancy, the patient group consisted of female patients and their male partners experiencing idiopathic RM (≥3 consecutive miscarriages before week 22 of gestation without any identified cause). In total, the current study included 558 RM patients from Estonia (80 female and 39 male partners) and Denmark (229 female and 210 male cases) (Supp. Table S1). For all recruited cases, known clinical risk factors of RM have been excluded (Supp. Materials and Methods). The female patients were further subphenotyped as either primary (Estonia, *n* = 46; Denmark, *n* = 113) or secondary RM (Estonia, *n* = 34; Denmark, *n* = 116) based on the occurrence of consecutive miscarriages either before (if any) live births or following one or more live births, respectively.

The control group consisted of 205 fertile females from Estonia (*n* = 90) and Denmark (*n* = 115) with no history of miscarriages and at least two (Denmark) or three (Estonia) successful pregnancies ending with live birth (Supp. Table S1). Male partners of fertile female controls were not included into the study due to limited information on their previous reproductive history.

#### Population-based cohort samples from Estonian Biobank (EGCUT)

The carrier status of the identified RM-associated risk CNV at 5p13.3 was additionally determined for population-based cohort samples (*n* = 1,000; 504 men, 496 women) drawn from the Estonian population-based Biobank (EGCUT; www.biobank.ee; Supp. Materials and Methods) and previously subjected to SNP genotyping with Human370CNV-Duo SNP array (Illumina Inc., San Diego, CA) [Nelis et al., 2009] and genome-wide CNV calling (Priit Palta, unpublished data).

### Discovery Phase: Genome-Wide SNP Genotyping and CNV Detection

The study design is schematically outlined in Figure1. In the discovery phase, proportionally one-third subsample (*n* = 70; fertile controls, *n* = 27; RM cases, *n* = 43; Supp. Table S2) of all Estonian study subjects (*n* = 209) was genotyped using Illumina Human370CNV-Quad SNP array (Genotyping Core Facility, Estonian Biocentre). For each sample, calling of CNVs from the resulting genome-wide genotyping data was performed in parallel with two algorithms, QuantiSNP and PennCNV [Colella et al., 2007; Wang et al., 2007] (Supp. Materials and Methods). The initial CNV calls from the two algorithms were merged and only CNVs that were called by both algorithms for the same individual in the same genomic loci were considered in the subsequent global analysis. Discrete CNV regions (CNVRs) were defined by merging overlapping CNV calls across all individuals in a study group as described previously [Redon et al., 2006; Perry et al., 2008]. Genomic distribution of identified CNVRs was visualized using Circos software (http://circos.ca/) [Krzywinski et al., 2009]. The genome-wide CNV data of this study have been submitted to the Database of Genomic Variants (DGV; http://projects.tcag.ca/variation/).

For the EGCUT population-based cohort samples, the microarray data were processed in identical manner using parallel analysis of QuantiSNP and PennCNV and by considering only CNV calls that were made by both algorithms. Accurate CNV copy number for the 5p13.3 duplication locus was drawn from the QuantiSNP data due to its higher precision in copy number estimation in this locus.

### Functional Enrichment Analysis

In order to acquire the up-to-date genomic annotation data for functional enrichment analysis, all CNVR breakpoint coordinates were converted to the latest version of the human reference sequence (from NCBI36/hg18 to GRCh37/hg19) (Supp. Materials and Methods). The list of genes within the identified CNV regions (extended by 10 kb on either side of the CNVR) was then acquired from the Ensembl database (version 69; http://www.ensembl.org/index.html). Functional enrichment analysis of subsequent gene sets was carried out separately for fertile controls and RM patients using g:Profiler gGOSt web-based software (http://biit.cs.ut.ee/gprofiler/) [Reimand et al., 2007, 2011] (Supp. Materials and Methods). Two outlier cases with increased genomic burden of CNVs (RM-M45 and RM-F4) were excluded from the analysis to avoid biased results. Results of Gene Ontology (GO) and Reactome (REAC) datasets with up to third relative hierarchy level were taken into account and enrichment for functional terms was considered significant if the multiple testing corrected enrichment *P*-value was <0.05.

### Prioritization of CNVRs for Experimental Confirmation and Subsequent Analysis

Discrete CNV regions identified in the discovery phase based on the whole genome SNP array genotyping data (full list of CNVRs in Supp. Table S3) were selected for experimental confirmation and further analysis if the following criteria were met: CNVR was (1) present in >1 individual (criterion met by 118 out of 423 nonoverlapping CNVRs), (2) found only among RM patients or overrepresented in RM patients with odds ratio (OR) ≥1.5 (45/118 CNVRs), and (3) intersected with or located in the proximity (up to approximately 200 kb) of biological candidate genes with a potential impact on the course of pregnancy based on previously published literature (9/45 CNVRs). As our study aimed to identify common rearrangements predisposing to RM, previous reports in the DGV database for the prioritized CNVRs of this study were considered as confirmation of true CNV loci, rather than as an exclusion criterion due to unknown reproductive success of the genotyped individuals in these reports.

### Experimental Copy Number Estimation of Prioritized CNVRs Using TaqMan qPCR

For experimental testing, TaqMan quantitative PCR (qPCR) was performed with one assay for seven of the prioritized CNVRs and with two assays in parallel for the two largest CNV loci as described in Supp. Materials and Methods (Supp. Table S4). Ten nanograms of genomic DNA was amplified using predesigned TaqMan copy number assays (Applied Biosystems, Foster City, CA; Supp. Table S5) or previously published TaqMan qPCR primers and FAM-tagged probe (6p21.33 CNVR; Supp. Table S6) [Parajes et al., 2007]. Copy number was normalized to the reference RNase P and population-specific pool of control DNAs (Supp. Materials and Methods). The diploid genomic copy number was calculated by multiplying the normalized TaqMan qPCR copy number estimates by two. Because of limitations of TaqMan qPCR assay to accurately determine very high diploid copy numbers, individuals with estimated locus copy number larger than four were assigned into copy number class “>4 copies per diploid genome.”

### Experimental Fine Mapping and Characterization of the 5p13.3 Rearrangement

The confirmation of the 5p13.3 duplication endpoints estimated by SNP array was performed with four EvaGreen qPCR assays (Supp. Table S6; Supp. Fig. S1) flanking the predicted breakpoints and using samples with known 5p13.3 copy number based on the data of TaqMan qPCR copy number typing (Supp. Materials and Methods). Copy number was estimated using absolute quantification method and normalized to the reference gene *ALB* and population-specific pool of control DNAs.

The exact position of duplication breakpoint junction in three 5p13.3 duplication carriers was determined using DNA sequencing by primer walking and targeting breakpoint junction region (5.8 kb) defined based on EvaGreen mapping (Supp. Materials and Methods). A control PCR spanning the identified breakpoint junction of the 5p13.3 tandem duplication (BP-PCR) was applied to confirm the duplication carriership in all Estonian and Danish RM case-control samples previously addressed with TaqMan qPCR (Supp. Materials and Methods).

The DNA sequences flanking the identified duplication breakpoints (±1,000 bp) were screened for repetitive elements using RepeatMasker software (http://www.repeatmasker.org/) and searched for the non-B DNA motifs including direct and inverted repeats, short-tandem repeats, and cruciform motifs using non-B DNA motifs search tool (http://nonb.abcc.ncifcrf.gov/apps/nBMST/default/).

### Expression Profile Analysis of *PDZD2* and *GOLPH3* Genes

The expression analysis was performed using human tissue cDNA panels Human MTC panel I and II (BD Biosciences Clontech, Palo Alto, CA) consisting of pools of samples for each tissue (Supp. Materials and Methods). The expression profile of the *GOLPH3* and *PDZD2* genes was determined with TaqMan qPCR approach using predesigned TaqMan gene expression assays (Applied Biosystems) and normalization to the reference transcript of *HPRT*.

### Copy Number Assignment of TaqMan qPCR Values

The copy number assignment for simple deletion and/or duplication polymorphisms was performed manually, whereas for the multicopy 5p13.3 locus, average copy number ratio of two TaqMan assays located within the rearranged region was used for the assignment of each sample into a distinct copy number cluster with k-means clustering method in the statistical package R (ver. 2.15.0; http://www.R-project.org/) (Supp. Materials and Methods). The resulting grouping was confirmed using EvaGreen qPCR performed with assay “EvaGr assay 2” located within the rearranged region (Supp. Fig. S1). Furthermore, the 5p13.3 duplication carriership was confirmed in all Estonian and Danish RM cases and fertile controls using the duplication junction-specific control PCR (BP-PCR). Subsequent groups of carriers versus noncarriers were subject to genetic association testing (Supp. Fig. S2).

### Genetic Association Testing

Genetic association of the 5p13.3 duplication with the clinical diagnosis of RM was tested using logistic regression model in the statistical package R (version 2.15.0) in the Estonian and Danish RM cases/fertile control sample sets with. Subjects were assigned as either carriers or noncarriers (wild type) of the tandem duplication. The obtained population-specific results were subsequently combined in the classical meta-analysis approach based on effect size estimates (*beta-*statistic) and standard errors and performed with the inverse-variance method under fixed-effects model in the statistical package R. Alternatively, to correct for the asymmetric case/control ratio in the Estonian and Danish sample sets, a z-score-based meta-analysis was performed combining the logistic regression *P*-values, effect directions, and effective sample sizes using METAL software as described by Willer et al. (2010). In Estonians, the RM patient group was further tested against Estonian Biobank population-based cohort samples using logistic regression analysis as described above. Results with *P*-values <0.05 were considered significant.

## Results

### Increased Genomic Burden of CNVs in Two RM Patients

In the discovery phase, the whole-genome screening (Illumina Human370CNV-Quad SNP array) for structural variants was performed in one-third subset of Estonian case-control samples (*n* = 70; Supp. Table S2). On average, 13.3 CNVs were determined per individual in RM cases (*n* = 43) and 12.6 CNVs in fertile female controls (*n* = 27). Among all detected CNVs (*n* = 915) in the full discovery sample, a 2.1-fold excess of deletions compared to duplications was observed consistent with previously reported ratios [Redon et al., 2006; Conrad et al., 2010]. However, the median length of duplications exceeded significantly the size of deletions (60.8 kb vs. 26.1 kb; Mann–Whitney *U* test, *P* = 1.82 × 10^−13^).

**Figure 2:**
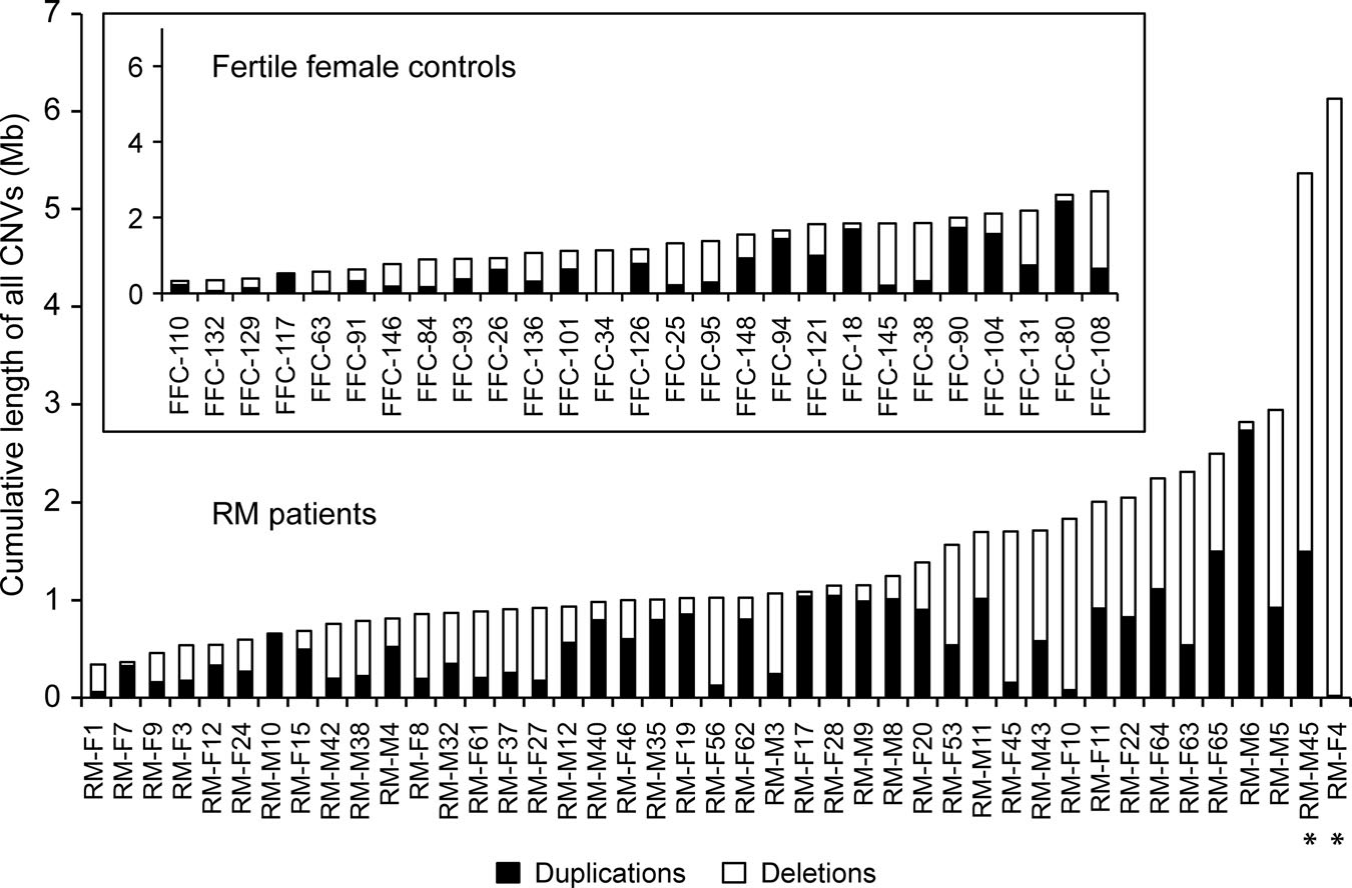
Genomic burden of all CNVs in the Estonian discovery phase sample set. Cumulative length of all deletions and duplications is presented per individual in RM patients (*n* = 43) and fertile controls (*n* = 27). The outlier cases with increased cumulative burden of all CNVs (Supp. Fig. S3) are indicated with asterisk. Female and male patients with identical number codes represent RM couples (e.g., RM-F45 and RM-M45). FFC, fertile female control; RM-F, female RM patient; RM-M, male RM patient.

Genomic burden analysis of all detected CNVs per individual revealed two outlier RM patients RM-F4 and RM-M45 with increased cumulative size of CNVs exceeding the rest of the RM cases more than fivefold (6.1 Mb and 5.4 Mb vs. median of 1.0 Mb, respectively) (Fig.2; Supp. Fig. S3). The case RM-F4 was the carrier of only heterozygous deletions (*n* = 19; Fig.2, Supp. Fig. S4A) with the majority being longer than 100 kb (*n* = 15; median 313 kb) and deleting altogether 50 genes. Remarkably, the 5.4 Mb of CNVs (in total, *n* = 29; Fig.2, Supp. Fig. S4A) in the case RM-M45 rearranged a total of 378 genes of which 113 duplicated and five deleted genes were located within the genomic region of *immunoglobulin heavy chain* (*IGH*) gene cluster at 14q32.33 potentially regulating the immune function during pregnancy. Additionally, heterozygous deletions of genes related to spermatogenesis (*SOHLH1*; MIM# 610224, *SPATC1*; MIM# 610874) [Goto et al., 2010; Suzuki et al., 2012] and known RM candidate genes (such as *C4A* (MIM# 120810), *C4B* (MIM# 120820), and *IGF2* (MIM# 147470)) [Laitinen et al., 1991; Ostojic et al., 2008] were identified in this male patient potentially increasing the individual's risk of RM disease. Both patients were from couples suffering from unexplained primary RM with no prior live births at the time of recruitment and had experienced three (RM-M45) or four (RM-F4) consecutive miscarriages occurring before gestational week 12. The available medical records for the male partner RM-M45 do not report any other diseases. During the postrecruitment period, the female patient RM-F4 carrying 50 hemizygous genes has experienced further miscarriages (*n* = 3) but also live births (*n* = 2) as a result of clinical management. Additionally, she has developed insulin-dependent diabetes mellitus and euthyroid goiters potentially also attributable to the large cumulative burden of deletions.

### Functional Profiling of Genes Disrupted by CNVRs Reveals Enrichment of Immune Signaling Pathways among RM Cases

The CNVs identified in the discovery phase clustered into 423 nonoverlapping CNVRs in RM cases and fertile controls that were uniformly distributed across the genome (Supp. Table S3, Supp. Fig. S4B). Functional profiling analysis of all genes disrupted by these CNVRs in either RM patient group (excluding two outlier cases with increased CNV burden) or among controls (1151 and 553 genes, respectively) was undertaken to identify pathways and processes significantly modulated by the rearrangements. In RM patients, the analysis specifically highlighted the role of impaired immune signaling, antigen binding, and immunoregulatory interactions at the fetomaternal interface in recurrent pregnancy loss (Table1). Notably, alterations in the REAC pathways of “innate immunity signaling” (9.3% of genes rearranged in the pathway, *P* = 9.15 × 10^−3^), “Complement cascade” (13.0% of genes, *P* = 1.11 × 10^−3^), and “Fc gamma receptors interact with antigen-bound IgG” (21.1% of genes rearranged by CNVs, *P* = 2.60 × 10^−4^) were specific to RM cases as none of the genes belonging to these categories were affected by structural variants in fertile controls (Table1).

**Table 1:** Pathway Analysis of Genes Affected by CNVs Identified by Genome-Wide Screening of Estonian Discovery Sample Set

Functional category			RM cases^a^		Controls
Type	ID	Name	Level	Corrected *P*-value^b^	Corrected *P*-value^b^
*Functional categories significantly enriched in RM cases*					
GO	0003823	Antigen binding	1	1.16 × 10^−4^	1.00
REAC	168256	Signaling in immune system	1	1.44 × 10^−3^	1.00
	168249	Innate immunity signaling	1.1	9.15 × 10^−3^	No genes^c^
	166658	Complement cascade	1.1.1	1.11 × 10^−3^	No genes^c^
	198933	Immunoregulatory interactions between a lymphoid and a nonlymphoid cell	1.2	1.61 × 10^−4^	1.00
	199161	Fc gamma receptors interact with antigen-bound IgG	1.2.1	2.60 × 10^−4^	No genes^c^
*Functional categories significantly enriched in fertile controls*					
GO	0060089	Molecular transducer activity	1	1.00	2.19 × 10^−11^
	0004871	Signal transducer activity	1.1	1.00	2.19 × 10^−11^
	0071944	Cell periphery	2	1.00	1.28 × 10^−7^
	0005886	Plasma membrane	3	1.00	4.17 × 10^−8^
	0044425	Membrane part	4	1.00	3.27 × 10^−6^
	0031224	Intrinsic to membrane	4.1	1.00	5.36 × 10^−6^
	0016021	Integral to membrane	4.1.1	1.00	5.36 × 10^−5^
	0016020	Membrane	5	1.00	3.90 × 10^−4^
	0004872	Receptor activity	6	1.00	1.16 × 10^−15^
	0038023	Signaling receptor activity	6.1	1.00	2.02 × 10^−13^
	0004888	Transmembrane signaling receptor activity	6.1.1	1.00	1.36 × 10^−14^
REAC	372790	Signaling by GPCR	1	1.00	5.60 × 10^−3^
	381753	Olfactory signaling pathway	1.1	1.00	9.99 × 10^−7^
	381750	Olfactory receptor-G protein olfactory trimer complex formation	1.1.1	1.00	9.99 × 10^−7^

The list of rearranged genes in either patients (*n* = 43) or controls (*n* = 27) was subjected to functional enrichment analysis using g:Profiler software (Reimand et al., 2007) and included Gene Ontology (GO) and Reactome (REAC) functional categories up to third relative hierarchical level. gProfiler performs statistical enrichment analysis to identify functional groups and/or biological pathways that are significantly overrepresented in the user-provided gene list.

a Two outlier cases RM-F4 and RM-M45 with increased genomic burden of CNVs (Fig.2) removed from the analysis.

b Multiple testing corrected enrichment *P*-value.

c None of the genes in this functional category were disrupted by CNVs among controls.

GPCR, G protein coupled receptor.

Among the controls, the analyzed CNVRs likely represent benign rearrangements as only biological pathways of general cellular function were identified, including signaling of olfactory receptors (“olfactory signaling pathway,” REAC:381753, *P* = 9.99 × 10^−7^), a large class of genetically diverse proteins in humans known to be affected by CNVs in healthy individuals [Wong et al., 2007; Hasin-Brumshtein et al., 2009; Mills et al., 2011].

### Experimental Testing of Prioritized CNVRs Detected by SNP Array

In order to identify common distinct CNV regions increasing the risk of RM, nine prioritized CNVRs were selected based on the genome-wide CNV screening results (Supp. Table S3; Supp. Materials and Methods) and subjected to copy number typing using TaqMan qPCR in the discovery sample set (*n* = 70) (see also Fig.1). Precise copy number estimation by TaqMan qPCR assays was observed for four prioritized CNVRs, identifying all CNV carriers predicted based on microarray analysis with no false positives (Supp. Table S4, Supp. Fig. S5; false negative rate across regions 1.4%). Three CNV regions— *IGKV* (*immunoglobulin kappa variable cluster* at 2p11.2; MIM# 146980), *DKK2* (*Dickkopf 2 homolog* at 4q25; MIM# 605415), and *PDZD2:GOLPH3* (*PDZ domain containing 2*; MIM# 610697; *golgi phosphoprotein 3* at 5p13.3; MIM# 612207)—were carried onto the next experimental stage to analyze the full Estonian RM case-control sample set using the established TaqMan qPCR approach (Fig.1). *SEPT14* CNVR (*septin 14* at 7p11.2; MIM# 612140) was excluded from the next stage due to its restricted testis-specific expression [Peterson et al., 2007].

Although confirmed as copy number variable by TaqMan qPCR, precise locus copy number estimation of the remaining five prioritized CNVRs (Supp. Table S4) was most likely hindered due to complex genomic architecture and rearrangements of the region as also evident based on previous CNV studies (Supp. Fig. S6). Thus, these CNVRs were excluded from further genetic association testing.

### Significantly Increased Prevalence of *PDZD2:GOLPH3* Duplication in Estonian and Danish RM Cases

**Figure 3:**
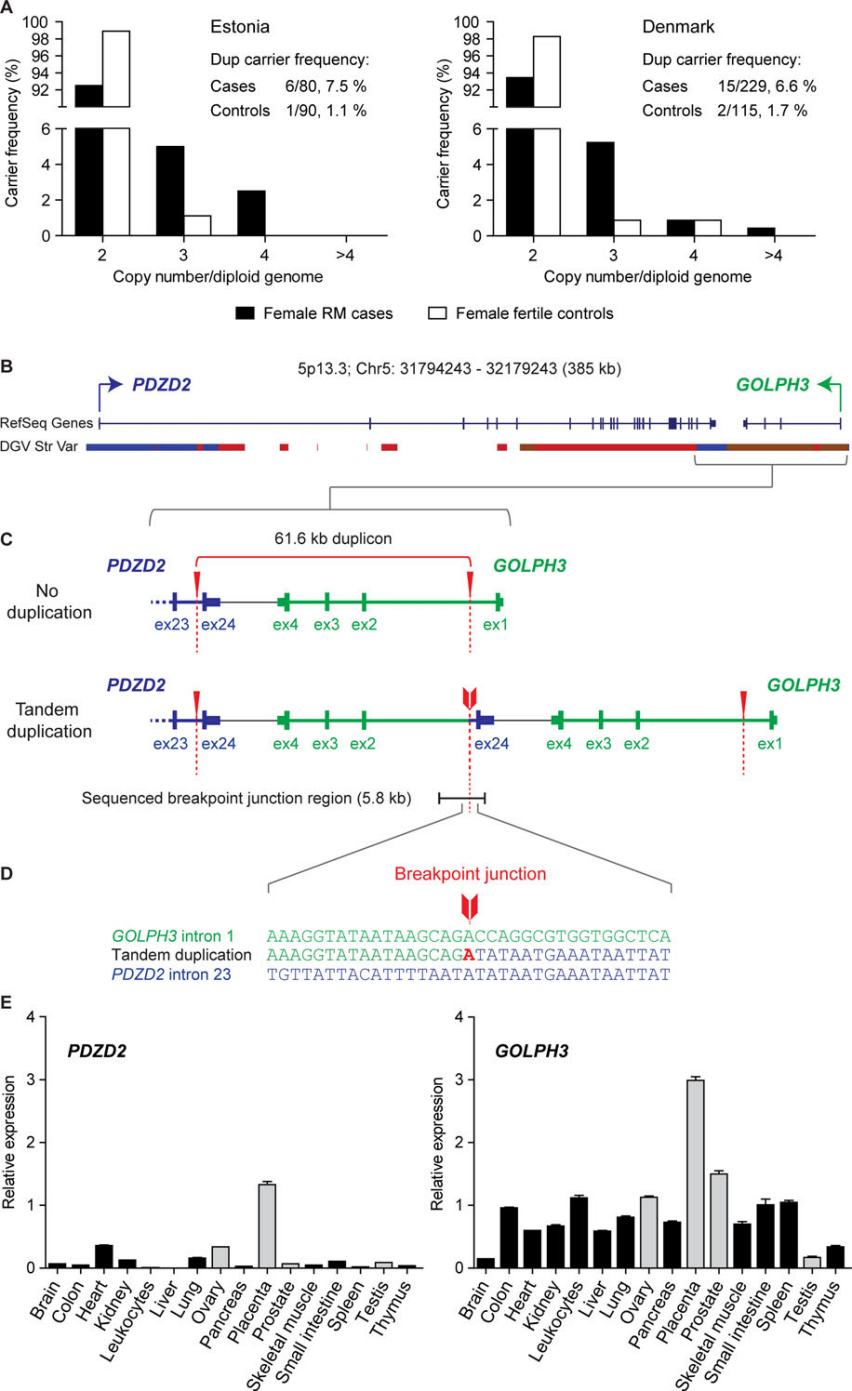
Copy number distribution of the *PDZD2:GOLPH3* duplication at 5p13.3 among female study subjects and experimental characterization of the locus. A: Copy number distribution of the *PDZD2:GOLPH3* CNV carriers and carrier frequency among female RM cases and female fertile controls from Estonia and Denmark. CNV carriers have three to four copies of the duplication per genome in Estonia and three to more than four copies per genome in Denmark (Supp. Fig. S2). Dup, duplication. B: Genomic context of 5p13.3 involving *PDZD2* and *GOLPH3* genes based on UCSC database (hg19). The opposite transcription of the *PDZD2* and *GOLPH3* genes is indicated with blue and green arrows, respectively. DGV Struc Var, structural variation data from the Database of Genomic Variants. C: Schematic representation of the 5p13.3 CNV locus with or without tandem duplication. Experimentally confirmed duplication endpoints are indicated with red arrowheads and dotted lines. The breakpoint junction of the tandem duplication is marked with red arrow tail and the breakpoint junction spanning region (5.8 kb) targeted with sequencing by primer walking is indicated with black bar. Ex, exon. D: Alignment of the acquired DNA sequence of the duplication breakpoint junction to the sequences of the proximal breakpoint region in *PDZD2* intron 23 and distal breakpoint region in *GOLPH3* intron 1. The microhomology of 1 bp determined at the junction of the duplication endpoints (red arrow tail) is shown with bold red letter. E: Gene expression profile of the *PDZD2* and *GOLPH3* genes in the human cDNA tissue panels. Expression level is given relative to the reference gene *HPRT* and as average of three amplification reactions ± SEM. Gene expression levels in reproductive tissues are highlighted with gray bars. Each tissue sample is compiled of a pool of cDNAs (range, *n* = 2–98; Supp. Materials and Methods) with *n* = 8 for placenta, *n* = 15 for ovary, *n* = 98 for prostate, and *n* = 45 for testis sample.

Among the three CNVRs (*PDZD2:GOLPH3* duplication, *IGKV* deletion/duplication, and *DKK2* deletion) tested in the full Estonian sample set (Supp. Table S1), *PDZD2:GOLPH3* duplication detected in up to four copies per diploid genome exhibited the strongest effect (OR = 7.28) with a higher prevalence of duplication carriers among the RM cases compared to fertile controls (9/119, 7.6% vs. 1/90, 1.1%, respectively) (Supp. Table S7). The Replication study of the *PDZD2:GOLPH3* CNVR performed in Danish RM cases and fertile controls (in total, *n* = 554; Supp. Table S1) confirmed the increased carrier frequency of the rearrangement (RM patients, 19/439, 4.3%; controls, 2/115, 1.7%; Table2). However, a sex-stratified analysis of the Danish RM patients revealed the higher prevalence only among the female patients (6.6%) that was comparable to Estonian female cases (7.5%) (Fig.3A, Table2). The *PDZD2:GOLPH3* duplication reached statistically significant association with an increased risk of RM (OR = 4.82, *P* = 0.012) in the meta-analysis combining the results of the Estonian and Danish female patient-control samples (in total, cases *n* = 309, controls *n* = 205). The high prevalence of the duplication observed among Estonian male RM partners was not replicated in Denmark (Supp. Table S8). The observed differences could be attributed to the different size of the Estonian and Danish sample sets (*n* = 208 and *n* = 558, respectively). These North European subjects have been recruited according to similar clinical criteria minimizing the interstudy heterogeneity and used in parallel in previous collaborative research [Nagirnaja et al., 2012; Rull et al., 2013].

**Table 2:** Maternal Risk of Recurrent Miscarriage Associated with the *PDZD2:GOLPH3* CNV at 5p13.3 in Estonia and Denmark

Women in association testing				Association testing^a^		
Controls	Number of subjects/carriers (%)	Female RM cases	Number of subjects/ carriers (%)	OR	CI (95%)	*P*-value
Association testing 1: full Estonian RM case-control sample						
Fertile women	90/1 (1.1)	All cases	80/6 (7.5)	7.22	0.85–61.25	0.070
		Primary RM	46/4 (8.7)	8.48	0.92–78.18	0.059
		Secondary RM	34/2 (5.9)	5.56	0.49–63.45	0.167
*Association testing 2: Estonian RM cases versus EGCUT cohort*						
EGCUT women	496/5 (1.0)	All cases	80/6 (7.5)	7.96	2.37–26.75	**7.9 × 10^−4^**
		Primary RM	46/4 (8.7)	9.35	2.42–36.15	**0.001**
		Secondary RM	34/2 (5.9)	6.14	1.15–32.88	**0.034**
*Replication study: Danish RM case-control sample*						
Fertile women	115/2 (1.7)	All cases	229/15 (6.6)	3.96	0.89–17.62	0.071
		Primary RM	113/9 (8.0)	4.89	1.03–23.15	**0.045**
		Secondary RM	116/6 (5.2)	3.08	0.61–15.60	0.174
*Meta-analysis: Estonian and Danish RM cases and fertile controls*						
Fertile women	205/3 (1.5)	All cases	309/21 (6.8)	4.82	1.42–16.40	**0.012**
		Primary RM	159/13 (8.2)	5.86	1.64–20.94	**0.007**
		Secondary RM	150/8 (5.3)	3.70	0.96–14.25	0.058

Association testing was performed with duplication carriers versus noncarriers as established based on TaqMan qPCR and confirmed by junction-spanning PCR for RM cases and fertile controls and estimated by QuantiSNP based on SNP genotyping and CNV calling data for EGCUT samples (Supp. Materials and Methods).

a Association testing was performed using logistic regression analysis in Estonia and Denmark separately. The results were subsequently combined in the meta-analysis using inverse-variance method under fixed-effects model. Results with *P*-value <0.05 were considered significant and are indicated in bold.

Notably, the strongest effect of the *PDZD2:GOLPH3* CNV was detected among the subgroup of women with primary RM (Estonian cases: *n* = 46, CNV carrier frequency 8.7%; Danish, *n* = 113, 8.0%) defined as ≥3 consecutive miscarriages and no preceding live births (meta-analysis, *n* = 159; OR = 5.86, *P* = 0.007) (Table2). A z-score-based meta-analysis correcting for the asymmetric case/control ratio provided consistent results in association testing including all female cases (*P* = 0.011) or only cases of primary RM (*P* = 0.006; Supp. Table S9).

The two CNVRs (*IGKV* locus, Del/Dup; *DKK2* locus, Del) with small differences in carrier frequencies in the full Estonian case-control sample (Supp. Table S7) likely represent benign common copy number variation with no major effect on the RM phenotype in our study.

### Low Prevalence of the *PDZD2:GOLPH3* Duplication in Worldwide Cohorts

The high risk of RM associated with the *PDZD2:GOLPH3* duplication was further confirmed when Estonian RM cases were compared to the Estonian Biobank (EGCUT) cohort samples (in total, *n* = 1,000) (Table2). The most significant association among the subgroups of cases was observed for female patients tested against female cohort subjects (*n* = 496; prevalence 1.0%; OR = 7.96, *P* = 7.9 × 10^−4^). Importantly, the carrier frequency of the *PDZD2:GOLPH3* duplication in the whole EGCUT cohort (9/1,000; 0.9%) was as low as observed among the Estonian and Danish controls (Table3) and similar carrier frequency has been consistently reported for various large worldwide population-based cohorts in the DGV (sample size range: *n* = 776–2,026; in total, *n* = 5846; duplication carrier frequency from 0.7% to 1.2%) (Table3).

**Table 3:** 5p13.3 CNV Carrier Frequency among Estonian and Danish Control Individuals of This Study and in the Populations Reported in the Database of Genomic Variants

	CNV	Number of					
	carriers	carriers/study			CNV	CNV size	
Population^a^	(%)	group	Phenotype	Detection method^b^	type	(kb)	Reference
*Dataset of this study*							
Denmark	1.7	2/115	Fertile controls	TaqMan qPCR	Dup	61.6	This study
Estonia	1.1	1/90	Fertile controls	Illumina Human370CNV-Quad, TaqMan qPCR	Dup	61.6	This study
Estonia	0.9	9/1,000	Cohort	Illumina Human370CNV-Duo	Dup	61.6	This study; Nelis et al. (2009)
*Dataset presented in the Database of Genomic Variants (DGV)*							
Ontario, Canada^c^	0.7	8/1,190	Cohort	Affymetrix 500K and 100K	Dup	35.1; 62^d^	Zogopoulos et al. (2007)
Worldwide (HGDP, NINDS)^e^	1.1	21/1,854	Cohort; neurological disease controls	Illumina HumanHap300, Illumina HumanHap240S, Illumina HumanHap650Y, Illumina HumanHap550	Dup	68.1	Itsara et al. (2009)
Worldwide (HapMap, PopGen)^f^	1.2	9/776	Healthy controls cohort	Affymetrix 500K	Dup	35.1; 95; 146^d^	Pinto et al. (2007)
Philadelphia, PA^c^,^g^	1.0	20/2,026	Healthy controls cohort	Illumina HumanHap550 V1	Dup	28.5; 38.9; 71.7^d^	Shaikh et al. (2009)

a In case of multiple studies targeting an identical study group (e.g., HapMap collection), results for the largest sample collection are presented.

b Only carrier frequencies from studies applying SNP array copy number estimations are included from the DGV database.

c Data for multiple overlapping nonrecurrent CNVs in 5p13.3 identified in the study are merged.

d CNV sizes of all overlapping CNVs in the region identified in the study.

e HGDP: cohort of the Human Genome Diversity Panel, 51 world populations, *n* = 1,064; NINDS, neurological disease controls of European descent from National Institute for Neurological Disorders and Stroke, *n* = 790.

f HapMap: healthy individuals from four populations—parent–offspring trios of the Yoruba from Nigeria (YRI; *n* = 30), parent–offspring trios of European descent from Utah (CEU; *n* = 30), unrelated Japanese from Tokyo, Japan (JPT; *n* = 45), and unrelated Han Chinese from Beijing, China (CHB; *n* = 45); PopGen: unrelated healthy individuals from Northern Schleswig-Holstein (Northern Germany), *n* = 506.

g The cohort included 1,492 unrelated individuals, 80 mother–father–child trios, 325 mother–child and 140 father–child duos, 59 siblings, and 10 twins.

Dup, duplication.

### Fine-Scale Experimental Mapping of the *PDZD2:GOLPH3* Duplication CNV

Experimental fine-scale mapping of the 5p13.3 duplication confirmed the extent of the CNV predicted by the discovery SNP microarray data from the last intron of the *PDZD2* to the first intron of the *GOLPH3* genes, which are transcribed from the opposite DNA strands (Fig.3B). Subsequent sequencing of the breakpoint junction-spanning region in the *PDZD2:GOLPH3* duplication carriers refined the exact genomic coordinates of the CNV as Chr5: 32106204 – 32167777 and length as 61.6 kb (compared to 52.4 kb based on SNP array estimation) (Fig.3C). The presence of identical recurrent tandem duplication was detected in all North European carriers of the *PDZD2:GOLPH3* CNV independent of the duplicon copy number.

The flanking genomic context of the *PDZD2:GOLPH3* duplication endpoints were characterized by a high number of repetitive elements in the proximity and overlapping with both breakpoint sites (Supp. Fig. S7). The proximal duplication endpoint was located within the DNA repetitive sequence *MER1A* and the distal endpoint in the *AluJb* element, whereas no extensive DNA sequence homology between the two repeat elements was observed. Also, no DNA sequences leading to non-B DNA conformation were identified and only a microhomology of one nucleotide was determined at the junction of the duplication endpoints by sequencing (Fig.3D). Thus, repeat-mediated rearrangement mechanisms other than nonallelic homologous recombination have possibly contributed to the occurrence of this recurrent duplication (reviewed in [Hastings et al., 2009]).

### Expression Analysis of *PDZD2* and *GOLPH3* Genes in Human Tissues Reveals the Highest Expression in Placenta

Although *GOLPH3* was ubiquitously expressed in all tested tissues (human MTC panels I and II), it exhibited the highest transcript level in the placenta (average expression relative to reference *HPRT*, 2.99 ± 0.06 SEM), followed by prostate, ovary, and leucocytes (relative expression 1.50 ± 0.05, 1.13 ± 0.02, and 1.12 ± 0.04; respectively) (Fig.3E). In general, the level of *PDZD2* transcripts was low in all tissues; however, the highest expression was also determined for the placenta exceeding fourfold the detected mRNA quantities in the two other sites of highest expression, heart and ovary (relative expression 1.33 ± 0.05 vs. 0.36 ± 0.01 and 0.34 ± 0.0, respectively) (Fig.3E). Overall, the expression profile of these genes points to their functional relevance in the placenta and potentially in other reproductive organs.

## Discussion

In this study, we have addressed the effect of genome-wide CNVs and contribution of selected CNV regions in modulating the predisposition to RM. The genome-wide CNV profiling revealed a significant enrichment of rearranged genes linked to innate immune signaling and immunoregulatory interactions within the RM study group, indicative of a potential effect on early pregnancy maintenance. As a main result, we report a novel duplication locus at 5p13.3 conferring high risk of RM among the female subjects of both Estonia and Denmark, and disrupting the *PDZD2* and *GOLPH3* genes predominantly expressed in placenta and associated with pregnancy maintenance for the first time.

The large cumulative burden of CNVs and specifically long deletions may modify an individual's predisposition to RM due to increased chances of disrupting specific key genes or pathways essential for early pregnancy maintenance. A case-by-case analysis performed in this study identified two RM cases RM-F4 and RM-M45 distinguished by heavy burden of CNVs and accumulation of several long deletions in RM-F4 (*n* = 15 out of 19 CNVs; Fig.2). Several potential or known RM candidate genes that may independently or synergistically elevate the risk of RM were rearranged within these cases, including immunomodulatory loci such as 119 genes from the *IGH* cluster and the previously reported RM-associated *C4A* and *C4B* genes [Laitinen et al., 1991]. A subset of patients may thus exhibit increased risk of RM disease attributable to excessive genomic burden of CNVs; however, the finding remains to be confirmed by future studies.

The detailed functional enrichment analysis of genes under CNVs within the RM study group confirmed the highly specific overrepresentation of pathways related to immune function and highlighted the processes most sensitive to CNV alterations in early pregnancy such as antigen binding, immunoregulatory interactions, innate immunity signaling, and complement cascade pathway associated with RM previously (Table1) [Li and Huang, 2009; Denny et al., 2013]. The CNV profile modifying the repertoire of presented antigens and alloimmune responses may affect the subtle balance of the immunological tolerance at the fetomaternal interface. As mother is carrying a semiallogeneic fetus expressing paternally inherited alloantigens, maternal immune rejection has been implicated in the etiology of preeclampsia and RM previously [Wilczynski, 2006; Guleria and Sayegh, 2007; Faridi and Agrawal, 2011]. The major contribution of fetomaternal immune function in the development of RM has also been demonstrated in gene expression profiling of the maternal decidual tissue and chorionic villi of aborted fetuses [Baek et al., 2002; Krieg et al., 2012].

As a major outcome of this study, a common multicopy duplication at 5p13.3 was identified conferring increased maternal risk to RM in North European populations, Estonians and Danes (meta-analysis OR = 4.82, *P* = 0.012). The prevalence of the *PDZD2:GOLPH3* CNV was detected with more than fivefold higher frequency in the female RM patients compared to fertile women (6.6%–7.5% vs. 1.1%–1.7%) or worldwide population cohorts (0.7%–1.2%; Tables2 and 3). The largest effect of *GOLPH3:PDZD2* duplication as a risk factor was detected among the subgroup of women diagnosed with primary RM (meta-analysis, OR = 5.86, *P* = 0.007; Table2). The duplication breakpoints of the identified *PDZD2:GOLPH3* recurrent CNV (61.6 kb in size) occurring in up to >4 diploid copies (Fig.3A) were positioned within the *PDZD2* and *GOLPH3* genes transcribed in the opposite directions (Fig.3C). Although the 5p13.3 duplication does not directly alter the copy number of entire coding regions of the genes, the modifications in the local genomic context may nevertheless interfere with the transcription leading to dysregulation of the involved or neighboring genes as reported previously [Henrichsen et al., 2009a, 2009b]. The potential functional relevance of the identified rearrangement in pregnancy was supported by the expression profiling of *PDZD2* and *GOLPH3* in the human tissue panels revealing mutually high transcription levels in the placenta (two to fourfold increase compared to other tissues) but also in the maternal reproductive organ ovary (Fig.3E). We speculate that the significantly increased predisposition to RM among the female duplication carriers may be attributed to the joint effect in maternal reproductive tissues and in the placental tissue carrying a maternally inherited duplication CNV. The mechanism of action and joint effect of the 5p13.3 CNV remains to be addressed further due to current limited availability of ovarian and early miscarriage placental samples carrying the 5p13.3 CNV.

Most importantly, a recent study addressing the contribution of maternal CNVs in cases of preeclampsia (PE)—a severe late pregnancy disorder originating from placental dysfunction—identified the *PDZD2:GOLPH3* duplication as the genetic risk factor of the disease [Zhao et al., 2013]. The reported 50.4 kb duplication colocalized with the CNV identified in the current study and occurred with the prevalence of 8.3% among the affected and 1.6%–1.9% among unaffected female subjects. The identified risk to PE (OR = 4.80, *P* < 0.05) is close to the effect detected for the RM susceptibility in this study (Table2). The possible common origin of various pregnancy disorders has been acknowledged previously, including overlapping causality of RM and PE [Li and Huang, 2009; Baig et al., 2013]. Mutations in the dosage-sensitive gene *GLUT3* (MIM# 138170) have been reported as the common genetic risk factor leading to either early pregnancy loss or fetal growth restriction [Ganguly et al., 2007]. Thus, it is plausible that the identified *PDZD2:GOLPH3* duplication may represent a pleiotropic risk factor in the genetic etiology of not only RM but also other pregnancy complications.

Neither of the novel RM-associated genes *PDZD2* or *GOLPH3* has been linked to placental function or pregnancy maintenance previously. The function of PDZD2 is poorly defined and has mainly been addressed as a tumor suppressor [Yeung et al., 2003; Tam et al., 2006]. More extensively studied conserved GOLPH3 is essential for Golgi trafficking and maintenance of its structure [Dippold et al., 2009; Wood et al., 2012]. Recently, genomic amplification of the *GOLPH3* gene was linked to oncogenic features [Scott et al., 2009; Wang et al., 2012; Hu et al., 2013] and was shown to activate the signaling pathway of mechanistic target of rapamycin (mTOR), an essential component of mammalian reproductive function [Murakami et al., 2004; Wen et al., 2005; Busch et al., 2009; Yu et al., 2011; Gonzalez et al., 2012; Tanwar et al., 2012]. Alterations in mTOR signaling have been associated with multiple reproductive disorders in mice and human, including RM [Roos et al., 2007; Adhikari et al., 2010; Hirota et al., 2011; Leconte et al., 2011; Vatin et al., 2012], thus providing the potential functional link between the amplification of *GOLPH3* and development of RM disease.

In summary, the findings of this study highlight the genetic heterogeneity of RM not only by characterizing the potential predisposing effect of the cumulative burden of genome-wide CNVs, but also by identifying a common *PDZD2:GOLPH3* duplication, as RM risk factor detected in two independent North European sample sets. Importantly, the identified *PDZD2:GOLPH3* duplication may represent a novel genetic risk factor for several pregnancy complications. The underlying functional effect of the *PDZD2:GOLPH3* duplication in reproductive organs, placenta, and potentially in tumorigenesis remains to be addressed.
