# Supplementary material for: Structural Genomic Variation as Risk Factor for Idiopathic Recurrent Miscarriage
Source: Hum Mutat. 2014 Jun 24;35(8):972–82. doi: 10.1002/humu.22589 (PMC4285182; doi:10.1002/humu.22589)
Supplement: Supplementary file 1 [file humu0035-0972-SD1.pdf]

Supporting Information

Supp. Materials and Methods

Supp. Figure S1. Fine-mapping of the *PDZD2:GOLPH3* duplication endpoints at 5p13.3.

Supp. Figure S2. Distribution of unrounded copy number estimations for *PDZD2:GOLPH3* duplication based on TaqMan qPCR in Estonian and Danish RM cases (n = 119 and n = 439, respectively) and fertile controls (n = 90 and n = 115, respectively).

Supp. Figure S3. Cumulative length of all CNVs per individual among the discovery phase Estonian RM cases (n = 43) and fertile controls (n = 27).

Supp. Figure S4. Circos plots with distribution of copy number variable regions across the genome among the Estonian discovery samples.

Supp. Figure S5. Comparative copy number estimations for prioritized CNV regions predicted based on SNP microarray genotyping data and precisely determined with TaqMan qPCR in the Estonian discovery sample (n = 70).

Supp. Figure S6. Previously reported CNVs and segmental duplications for the complex prioritized CNV regions not carried on to the genetic association study in the full Estonian sample set (Supp. Table S4).

Supp. Figure S7**.** Genomic context flanking the 5p13.3 duplication endpoints in the *PDZD2* and *GOLPH3* genes.

Supp. Table S1. Characteristics of study subjects.

Supp. Table S2. Characteristics of Estonian study subjects analyzed in the discovery phase (n = 70).

Supp. Table S3. Summary of copy number variable regions identified based on Illumina SNP-array genotyping and CNV calling in Estonian discovery phase sample set (n = 70).

Supp. Table S4. Prioritized CNVRs predicted based on SNP array and subjected to experimental copy number typing using TaqMan qPCR assays in the discovery sample set.

Supp. Table S5. Pre-designed TaqMan Copy Number Assays (Applied Biosystems, Foster City, CA, USA) used in the study.

Supp. Table S6. Sequences of primers used in the study.

Supp. Table S7. Prevalence of the 2p11.2, 4q11.2 and 5p13.3 CNVs in Estonian RM cases and fertile controls.

Supp. Table S8. Sex-stratified association analysis of the *PDZD2:GOLPH3* CNV at 5p13.3 with recurrent miscarriage in Estonia and Denmark.

Supp. Table S9. Z-score based meta-analysis in the Estonian and Danish case-control sample.

Supp. Materials and Methods

Study subjects

Recurrent miscarriage cases and fertile control samples

The study subjects from Estonia were recruited at the Women's Clinic of Tartu University Hospital and Nova Vita Clinic, Tallinn, Estonia since 2003. The Danish subjects have been recruited at the Danish Recurrent Miscarriage Clinics, Copenhagen and Aalborg, Denmark since 1986. In total, the current study included 558 RM patients (80 female and 39 male partners from Estonia; 229 female and 210 male cases from Denmark) (Supp. Table S1). All recruited patients had a normal karyotype tested from peripheral blood lymphocyte cultures and known clinical risk factors of RM had been excluded. Female patients had normal menstrual cycles, no uterine anomalies (by hysterosalpingography, hydrosonography or hysteroscopy) and no antiphospholipid syndrome. The female patients were further sub-phenotyped as either primary (Estonia, n = 46, median age 29.5 years, 5 - 95th% 20.3 - 36.8 years; Denmark, n = 113, 29.0 years, 22.0 - 36.8 years) or secondary RM (Estonia, n = 34, median age 31.0 years, 5 - 95th% 23.0 - 37.0 years; Denmark, n = 116, 30.0 years, 23.8 - 39.0 years) based on the occurrence of consecutive miscarriages either before (if any) live births or following one or more live births, respectively.

The study was conducted according to the Declaration of Helsinki principles and a written informed consent was obtained from each individual prior to recruitment and collection of blood samples for DNA extraction.

Population-based cohort samples from Estonian Biobank (EGCUT)

The carrier status of the identified RM-associated risk CNV at 5p13 was additionally determined for population-based cohort samples (n = 1000; 504 men, 496 women) drawn from the Estonian Biobank. The cohort (18 years of age and up) closely reflects the age, sex and geographical distribution of the Estonian population. Studies on the genetic structure of Europe have placed Estonia in the proximity of Northern and Eastern European populations (Nelis et al., 2009). As the EGCUT study protocol incorporates information on self-reported experience of spontaneous miscarriages of the recruited subjects and does not include confirmed medical diagnosis of recurrent miscarriage disease, the self-reported data was not addressed in this study. The entire project is conducted according to the Estonian Gene Research Act and all of the participants have signed the broad informed consent.

Discovery phase: genome-wide SNP genotyping and CNV detection

In the discovery phase, proportionally one-third of Estonian sample (n = 70; fertile controls, n = 27; RM cases, n = 43; Supp. Table S2) was genotyped using Illumina Human370CNV-Quad SNP array (Illumina) (Genotyping Core Facility, Estonian Biocentre) with SNP call rate >99.4% for all samples (median 99.8%). For each sample, calling of CNVs from the resulting genome-wide genotyping data was performed in parallel with two Hidden Markov Model-based algorithms. Normalized signal intensity data was analysed with QuantiSNP program (Colella et al., 2007) calling CNVs independently for each individual. The adjustment for 'genomic waves' in signal intensities (Diskin et al., 2008) was turned on with the '--doGCcorrect' key and '--emiters 25' together with '--Lsetting 1000000' were added to the default calling parameters. A parallel analysis was performed with the same genotyping data applying PennCNV algorithm (Wang et al., 2007) and using an Estonian population specific B-allele frequency file (PFB file, calculated from 1000 EGCUT samples) as a reference dataset (Nelis et al., 2009). The default parameters and adjustment of genomic waves in signal intensities was applied as in QuantiSNP analysis. The initial CNV calls from the two algorithms were merged and only CNVs that were called by both algorithms for the same individual in the same genomic loci were considered in the subsequent analysis. As demonstrated previously, the advantage of using the intersection of CNV predictions by more than one algorithm effectively minimizes the number of false positive calls (Winchester et al., 2009; Pinto et al., 2011; Kim et al., 2012). CNVs with QuantiSNP log Bayes Factor (LBF value) <5 and/or rearrangements shorter than 250 bp were excluded from the resulting list of CNVs. For the EGCUT population-based cohort samples the microarray data was processed in a similar manner using parallel CNV calling by QuantiSNP and PennCNV.

Conversion of genomic coordinates

Following the CNV detection in the discovery phase and descriptive statistics of identified CNVs and CNVRs, we transferred all CNVR breakpoint coordinates from the reference genome assembly (NCBI36/hg18) to the latest version of human reference sequence (GRCh37/hg19) in order to acquire the up-to-date genomic annotation data. Conversion of coordinates was performed with the liftOver web-based batch conversion tool (http://genome.ucsc.edu/cgi-bin/hgLiftOver) and using default parameters. For 15 regions in total, the conversion was not possible, as these regions were duplicated, deleted or split in the newer version of the human reference genome assembly.

Functional enrichment analysis

The list of genes disrupted by CNVRs was acquired for the sample set of RM patients as well as for fertile controls using Ensembl PERL API to query the Ensembl database (version 69; http://www.ensembl.org/index.html). The query was extended by 10 kb on either side of the CNVRs in order to account for possible effects on the regulatory regions within immediate proximity of the involved genes (Pinto et al., 2010). Functional enrichment analysis of subsequent gene sets was carried out separately for fertile controls and all RM patients using g:Profiler gGOSt web-based software (http://biit.cs.ut.ee/gprofiler/) (Reimand et al., 2007; Reimand et al., 2011). Considering all genes in user-provided gene lists or chromosomal regions, g:Profiler performs statistical gene set enrichment analysis to find which functional groups and/or biological pathways are significantly overrepresented among user-provided genes, often helping with biological interpretation of high-throughput experiments. Results of Gene Ontology (GO) and Reactome (REAC) datasets with up to third relative hierarchy level were taken into account and enrichment for functional terms was considered significant if the multiple testing corrected enrichment P-value was <0.05. A reciprocal functional enrichment analysis in either RM cases or fertile controls was undertaken for functional terms specifically enriched in one of the study groups to determine the group-specific level of enrichment.

Experimental copy number estimation of prioritized CNVRs using TaqMan qPCR

In order to define the DNA region within the prioritized CNVRs most informative for experimental copy number estimation and placement of TaqMan qPCR assays, the genomic range of each selected rearrangement was narrowed down by identifying the minimal region overlapping between the CNV carriers based on the SNP array CNV calling data. For experimental testing, seven of the prioritized CNVRs (sized 294 bp to 49.6 kb; median 10.4 kb) were targeted with one TaqMan qPCR assay, whereas two largest CNV loci, a 52.4 kb duplication at chromosomal position 5p13.3 and 84.1 kb deletion at 14q32.33, were addressed with two assays in parallel (Supp. Table S4). DNA copy number typing with TaqMan quantitative PCR (qPCR) was performed in duplex reactions in a volume of 10 µl and consisting of 10 ng of genomic DNA, HOT FIREPol Probe qPCR Mix Plus (ROX) (Solis Biodyne, Tartu, Estonia), Copy Number Reference Assay RNAse P (Applied Biosystems, Foster City, CA, USA) and target-specific pre-designed TaqMan Copy Number assays (Applied Biosystems) as indicated in Supp. Table S5. CNV region at 6p21.33 involving the

CYP21A2 (MIM# 613815; Online Mendelian Inheritance in Man (OMIM) database; http://www.omim.org/) gene was targeted using previously published TaqMan qPCR primers and FAM-tagged probe (all 0.2 µM; Supp. Table S6) (Parajes et al., 2007) run together with 0.5 µl of VIC-tagged Copy Number Reference Assay RNAse P. All reactions were run in triplicate and detected on ABI Prism 7900HT Sequence Detection System (Applied Biosystems). Half of the samples on each plate were controls and half were RM patients in random order. Each plate included a population specific DNA pool of randomly selected fertile controls (n = 50) from either Estonia or Denmark. The copy number of a target region was determined using relative quantification method by normalization to the reference RNase P and the population-specific DNA pool. The diploid genomic copy number was calculated by multiplying the normalized TaqMan qPCR copy number estimates by two. Due to limitations of TaqMan qPCR assay to accurately determine very high diploid copy numbers, individuals with estimated locus copy number larger than four were assigned into copy number class '>4 copies per diploid genome'.

Experimental confirmation of the genomic position and range of 5p13.3 CNV

The confirmation of the 5p13.3 duplication endpoints estimated by SNP array was performed with four EvaGreen qPCR assays (EvaGr assays 1-4; Supp. Table S6; Supp. Figure S1) flanking the predicted breakpoints. Singleplex amplification reactions were run using 0.2 - 0.4 µM primers, HOT FIREPol® EvaGreen® qPCR Mix Plus (ROX) (Solis Biodyne) as per manufacturer's instructions and 10 ng of genomic DNA of samples with known 5p13.3 copy number based on the data of TaqMan qPCR copy number typing. Each copy number class (2, 3 or 4 copies per diploid genome) was represented by three individuals, except for the >4 copies/diploid genome identified in only one carrier. A reference gene *albumin* (*ALB;* MIM# 103600) was amplified in parallel with target loci for every sample under the same conditions and on the same plate (primers in Supp. Table S6). All reactions were run in triplicate and detected on ABI Prism 7900HT Sequence Detection System (Applied Biosystems). Copy number was estimated using absolute quantification method and normalized to the reference gene *ALB* and population-specific pool of control DNAs.

Fine-mapping of the 5p13.3 rearrangement

The exact position of duplication breakpoint junction in three 5p13.3 duplication carriers was determined using DNA sequencing by primer walking and targeting breakpoint junction region (5.8 kb) defined based on EvaGreen mapping. The sequencing was performed using

ABI Prism dGTP BigDye Terminator v3.0 Ready Reaction Cycle Sequencing Kit (Applied Biosystems) (junction-specific primers provided in Supp. Table S6). The obtained sequences were then aligned to the respective regions within the sequences of the *PDZD2* (MIM# 610697) and *GOLPH3* (MIM# 612207) genes (acquired from UCSC database; http://genome.ucsc.edu/cgi-bin/hgGateway) overlapping the duplication using ClustalW2 alignment (http://www.ebi.ac.uk/Tools/msa/clustalw2/).

A control PCR spanning the identified breakpoint junction of the 5p13.3 tandem duplication (BP-PCR) was applied to confirm the duplication carriership in all Estonian and Danish RM case-control samples previously addressed with TaqMan qPCR. The BP-PCR was performed using two 0.4 µM primers (Supp. Table S6) locating adjacent to the experimentally confirmed breakpoints within the rearranged region, 100 ng of genomic DNA and 1 U of HOT FIREPol DNA Polymerase (Solis Biodyne).

Expression profile analysis of *PDZD2* and *GOLPH3* genes

The expression analysis was performed using human tissue cDNA panels Human MTC panel I (Lot no 7080213) and Human MTC panel II (Lot no 8030311A) (BD Biosciences Clontech, Palo Alto, CA). The Human MTC panel I consists of human cDNA samples from brain (pool of n = 2 samples), heart (n = 3), kidney (n = 5), liver (n = 3), lung (n = 4), pancreas (n = 15), placenta (n = 8) and skeletal muscle (n = 7). Human MTC panel II is compiled of human cDNAs from colon (n = 5), ovary (n = 15), peripheral blood leucocytes (information not available), prostate (n = 98), small intestine (n = 32), spleen (n = 3), testis (n = 45) and thymus (n = 9). The expression profile of the *GOLPH3* and *PDZD2* genes was determined with TaqMan qPCR approach using pre-designed TaqMan Gene Expression Assays (Applied Biosystems). The total volume of the duplex amplification reactions was 10 µl and included 1 µl of cDNA, 0.5 µl of *HPRT* (MIM# 308000) TaqMan assay used as a reference, 0.5 µl of a target-specific TaqMan assay (*GOLPH3*, Hs00223239_m1; *PDZD2*, Hs01054836_m1) and HOT FIREPol Probe qPCR Mix Plus (ROX) (Solis Biodyne) as per manufacturer's instructions. All reactions were run in triplicate and detected using ABI Prism 7900HT Sequence Detection System (Applied Biosystems). Relative expression of target genes was determined by normalization to the reference transcript of *HPRT*.

Copy number assignment of TaqMan qPCR values

The copy number assignment for simple deletion and/or duplication polymorphisms at 2p11.2 and 4q25 CNVRs was performed manually due to clear clustering of TaqMan qPCR values to

distinct copy number classes (Supp. Figure S5). For the multicopy 5p13.3 locus, average copy number ratio of two TaqMan assays located within the rearranged region was used for the assignment of each sample into a distinct copy number cluster with k-means clustering method in the statistical package R (ver. 2.15.0; http://www.R-project.org/). The number of clusters to be used was inferred from the number of peaks in the averaged copy number distribution data (Supp. Figure S2). Three clusters (representing diploid copy numbers 2, 3 and 4) were used for the analysis of Estonian and four clusters (diploid copy numbers 2, 3, 4 and >4) for Danish samples.

SUPP. FIGURES

Supp. Figure S1. Fine-mapping of the *PDZD2:GOLPH3* duplication endpoints at 5p13.3. (A) Genomic context of 5p13.3 involving *PDZD2* and *GOLPH3* genes based on UCSC database (hg19). The opposite transcription of the *PDZD2* and *GOLPH3* genes is indicated with blue and green arrows, respectively. DGV Struc Var, structural variation data from the Database of Genomic Variants. (B) Mapping of duplication endpoints using TaqMan qPCR and EvaGreen qPCR assays. Yellow and red arrowed lines indicate the alternative duplication regions predicted by SNP array. Blue and green boxes in the schematic representation of genes denote the coding regions of *PDZD2* and *GOLPH3*, respectively, with exon numbers given above. Locations of qPCR assays are indicated with black arrows. Altogether, four EvaGreen qPCR assays (EvaGr assays 1 - 4) and one TaqMan qPCR assay in the *PDZD2* gene (Hs02781158_cn; Supp. Table S5) bordering with the predicted duplication breakpoints were used for experimental fine-mapping. Copy number values are given as mean ± SD of three representative individuals in each copy number class (2, 3 or 4 copies/diploid genome), except for copy number class of >4 copies with only one individual available for testing.

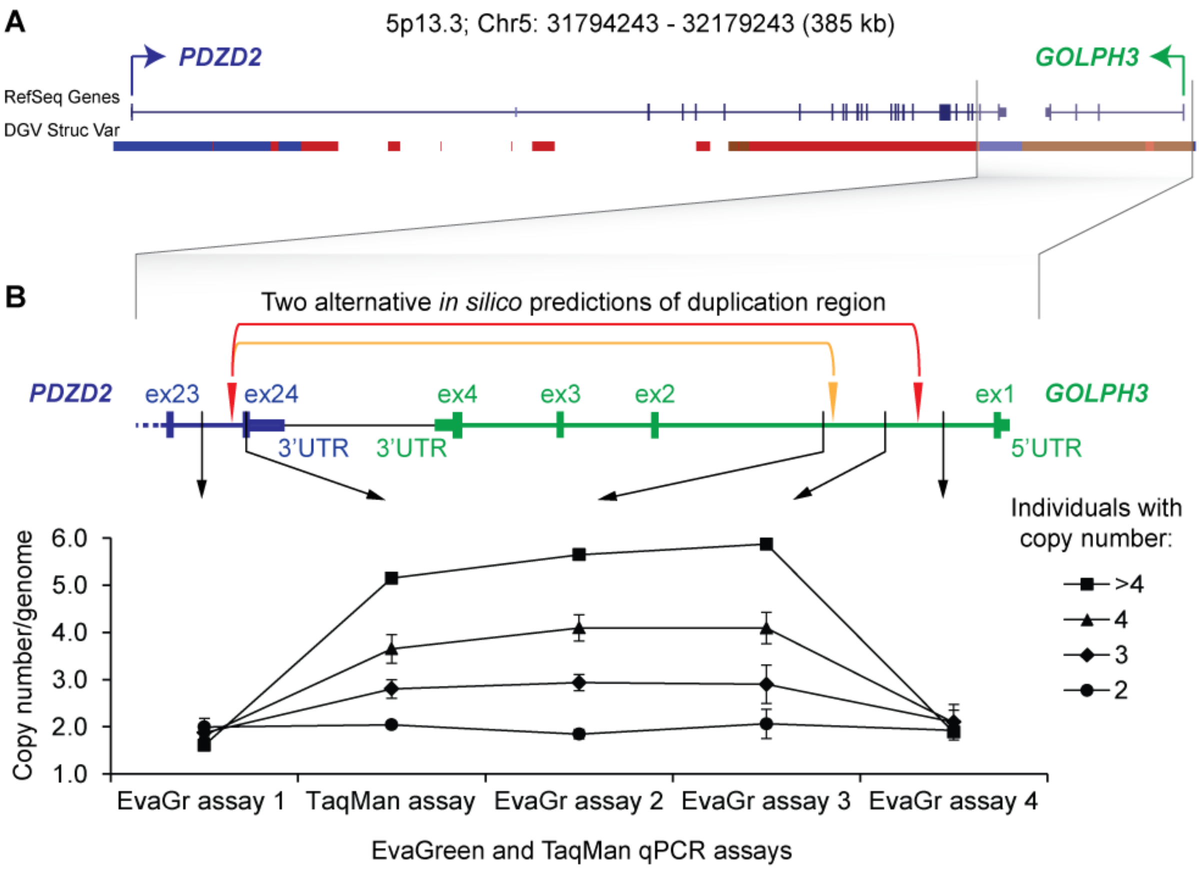

Supp. Figure S2. Distribution of unrounded copy number estimations for *PDZD2:GOLPH3* duplication based on TaqMan qPCR in Estonian and Danish RM cases (n = 119 and n = 439, respectively) and fertile controls (n = 90 and n = 115, respectively). All study subjects were further tested with the duplication junction-specific PCR (BP-PCR) to confirm the presence (indicated with green bars) or absence (black bars) of the tandem duplication. Based on this double estimation, all subjects were divided into subgroups of duplication carriers or non-carriers subsequently used in association testing. Separation of duplication carriers into distinct copy number classes (3, 4 or >4 copies per genome) is indicated (see also Supp. Materials and Methods). Due to limitations of TaqMan qPCR assay to accurately determine very high diploid copy numbers, individual with estimated locus copy number larger than four was assigned into copy number class '>4 copies per diploid genome'.

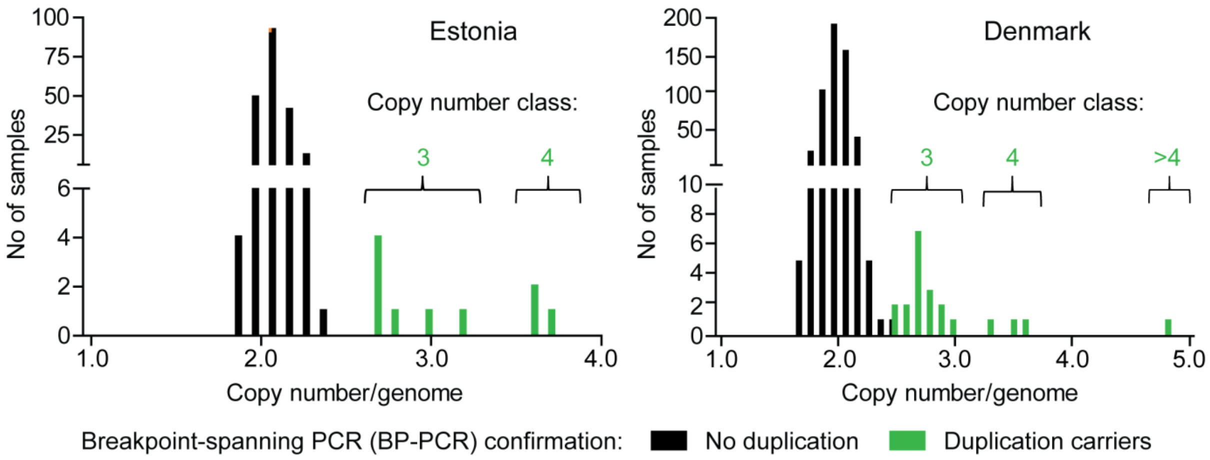

Supp. Figure S3. Cumulative length of all CNVs per individual among the discovery phase Estonian RM cases (n = 43) and fertile controls (n = 27). The boxes represent the 25th and 75th percentiles of the data, whereas the whiskers cover the extent of the data of up to 1.5x interquartile range. The median value is denoted as the line bisecting the boxes. Study subjects with outlier values are indicated with circles. RM, recurrent miscarriage; F, female; M, male.

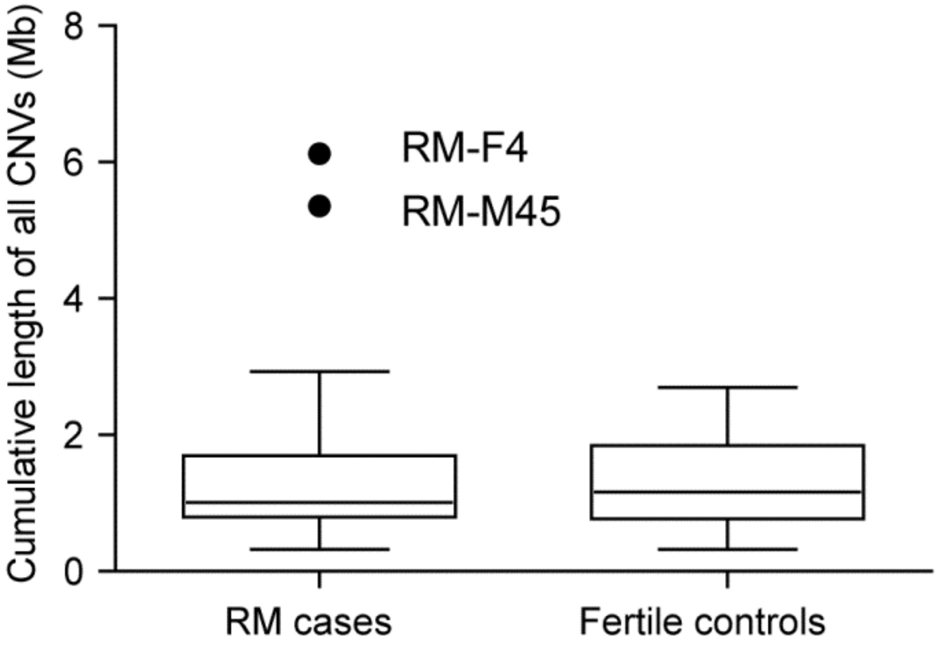

Supp. Figure S4. Circos plots with distribution of copy number variable regions across the genome among the Estonian discovery samples. (A) Genomic location of deletions and duplications for RM cases RM-F4 and RM-M45 with the largest cumulative burden of rearrangements in the genome. (B) Frequency and distribution of deletions and duplications among RM cases (n = 43) and fertile female controls (n = 27). The length of the bars (Y-axis) in the histogram represents the number of carriers of a CNV at the genomic locus, whereas the width correlates with the size of the CNVR. Single occurrence CNVs are highlighted in the yellow background. The number of carriers for outlier CNVRs of high frequency (>8 carriers in a study group) are indicated next to the respective bars.

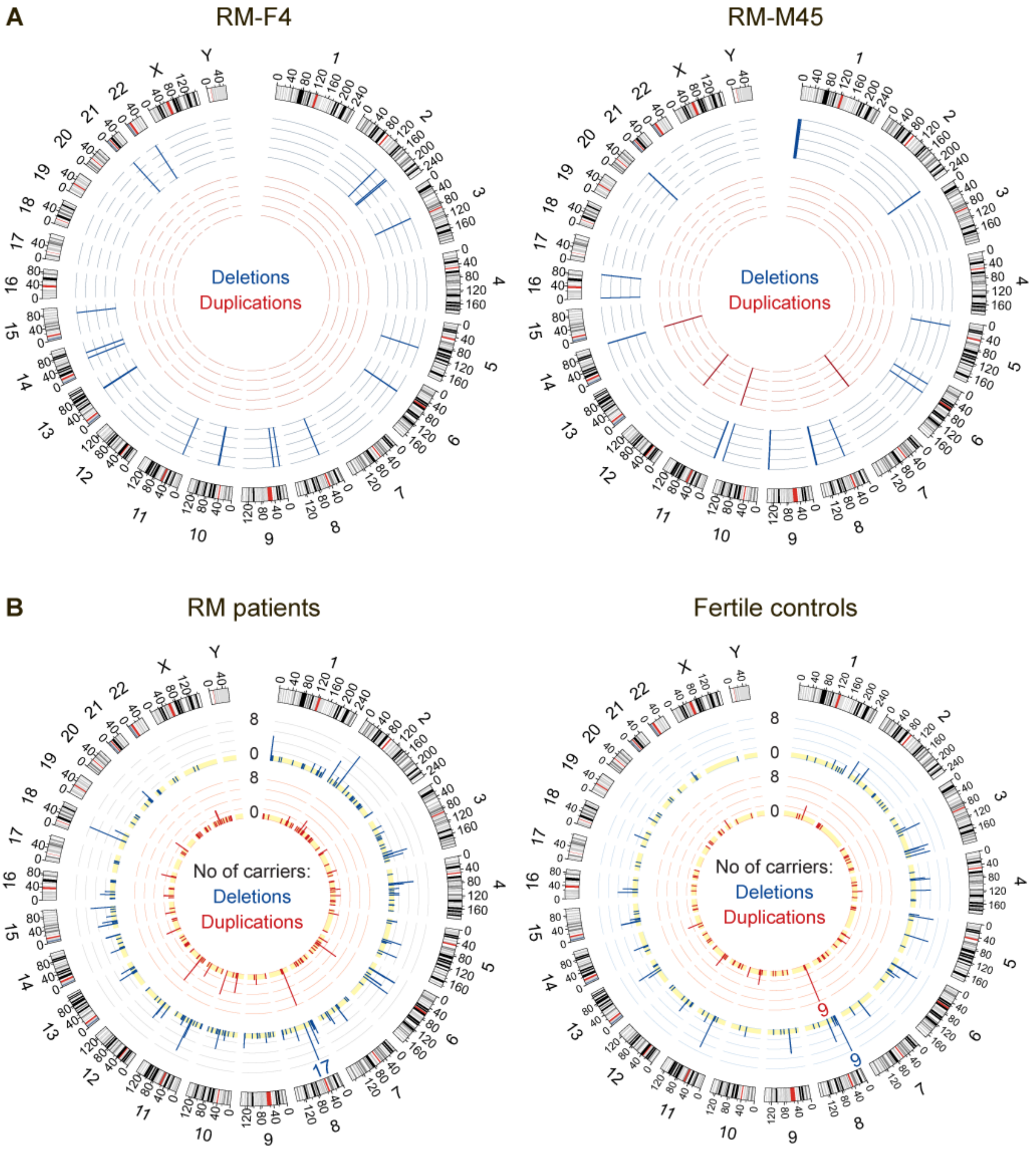

Supp. Figure S5. Comparative copy number estimations for prioritized CNV regions predicted based on SNP microarray genotyping data and precisely determined with TaqMan qPCR in the Estonian discovery sample (n = 70). SNP microarray prediction data is given according to QuantiSNP CNV calling results due to higher accuracy in copy number estimation compared to PennCNV in our dataset. Each CNV region was targeted with one TaqMan assay, except for the largest CNVR in the group, 5p13.3 (52.4 kb, predicted based on SNP array data), targeted with two assays within the locus (Supp. Table S5). Additional CNV carriers detected with TaqMan qPCR but unidentified by SNP microarray-based CNV calling are highlighted in red. TaqMan qPCR copy number values are unrounded and given per diploid genome.

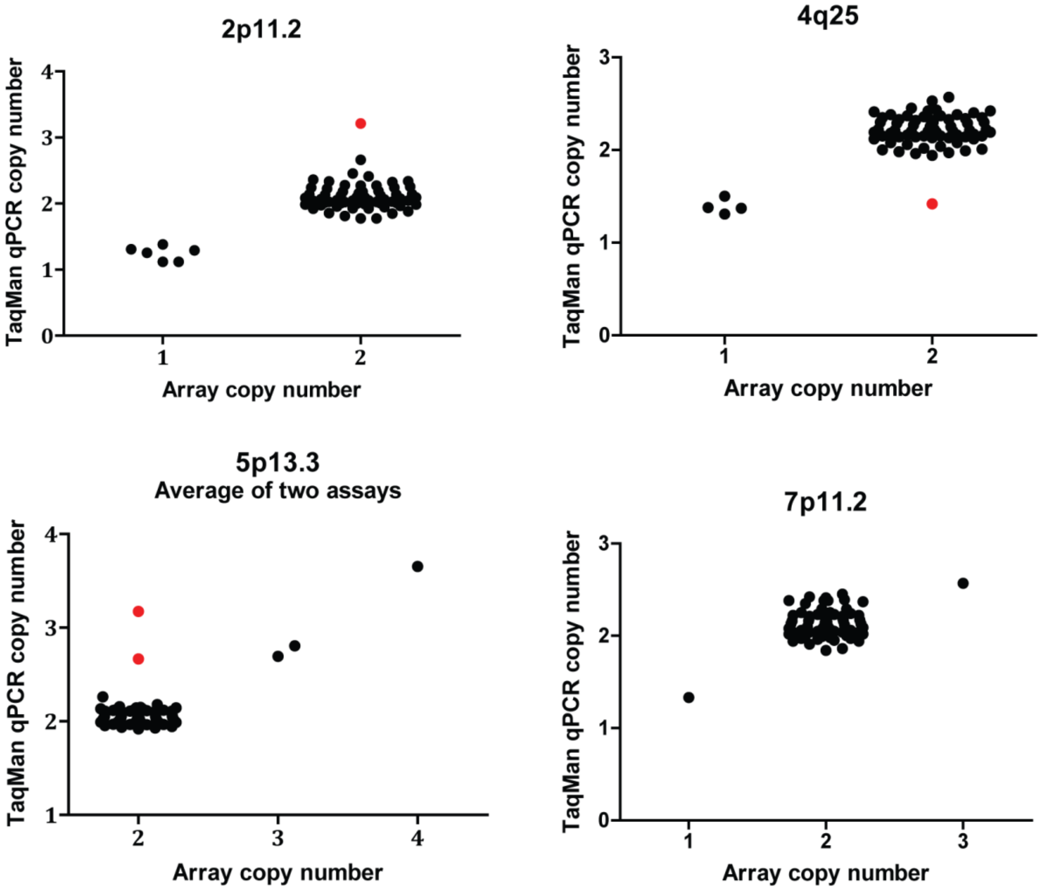

Supp. Figure S6. Previously reported CNVs and segmental duplications for the complex prioritized CNV regions not carried on to the genetic association study in the full Estonian sample set (Supp. Table S4). The CNV data is based on the Database of Genomic Variants (DGV) as presented in the UCSC browser (http://genome.ucsc.edu/cgi-bin/hgGateway). Chromosomal positions are given according to hg19. Red CNV tracks represent deletions, blue duplications, brown indicate both deletions and duplications in size relative to the reference and purple tracks denote inversions.

1. Chr1:1220136-1269723, deletion of 49587 bp, overlapping 6 genes.

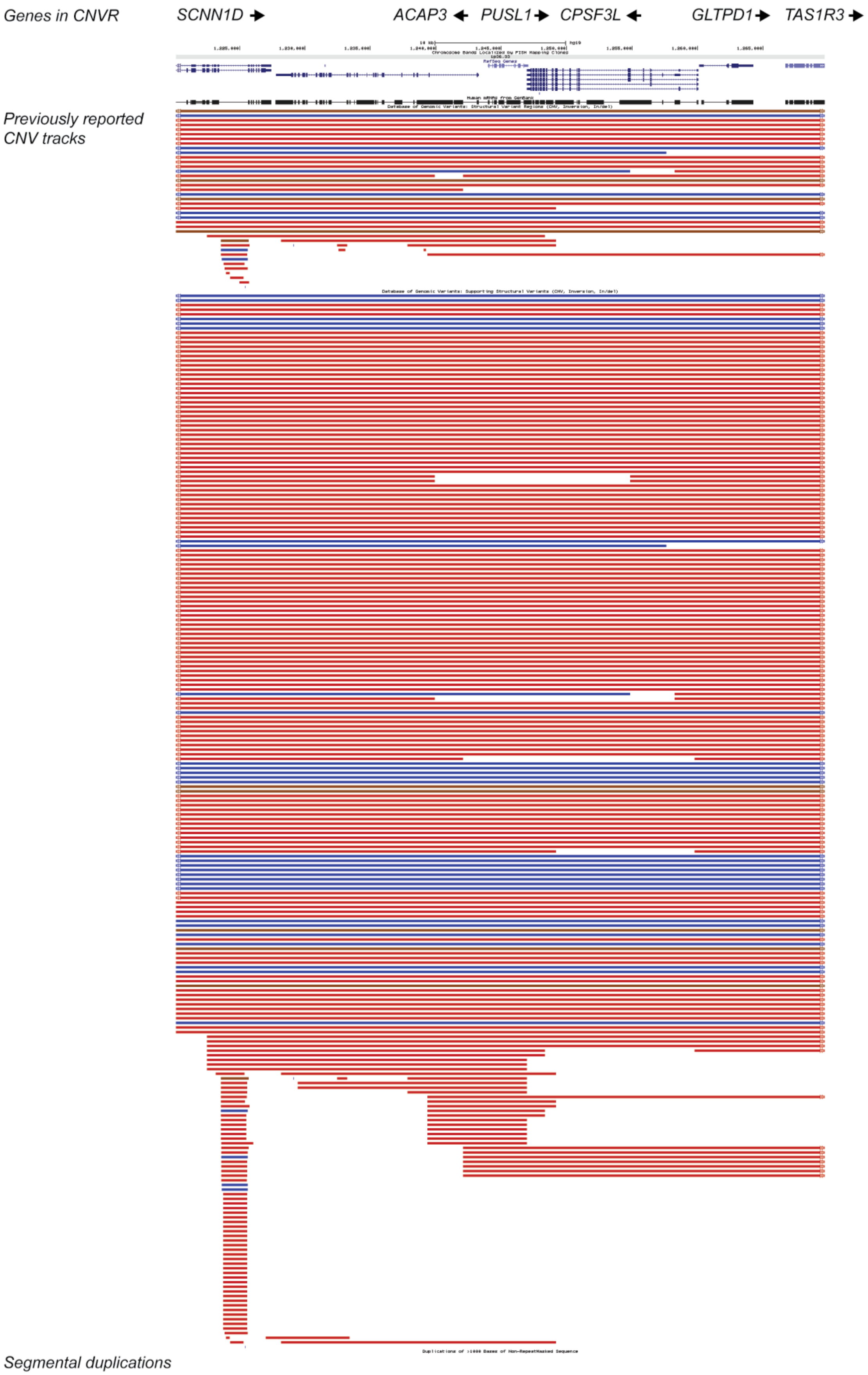

2. Chr6:32114582-32114875, deletion or duplication of 293 bp, overlapping *CYP21A2* gene.

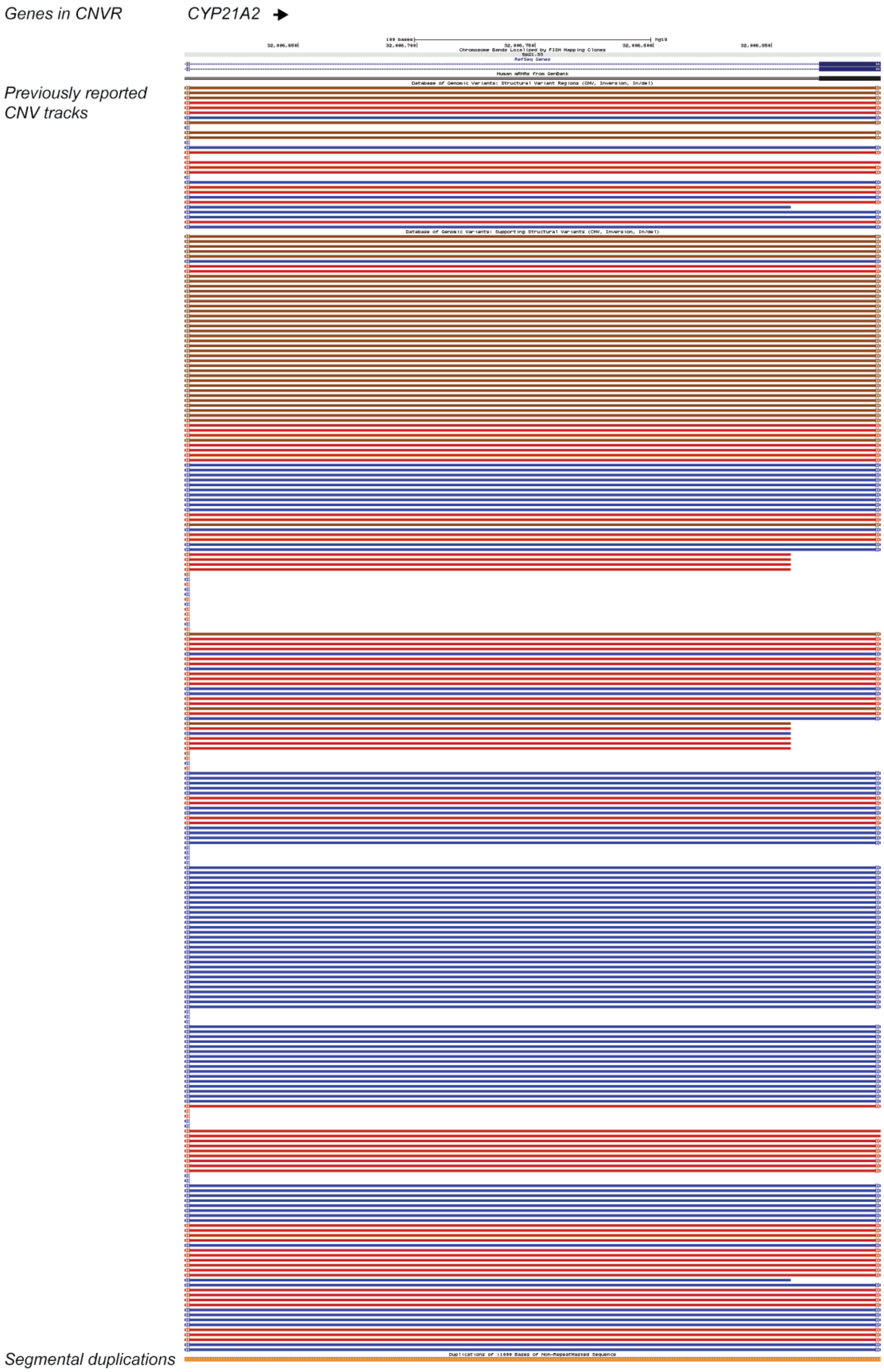

3. Chr8:16262509-16272892, deletion of 10383 bp, intergenic, 212 kb upstream from *MSR1* gene.

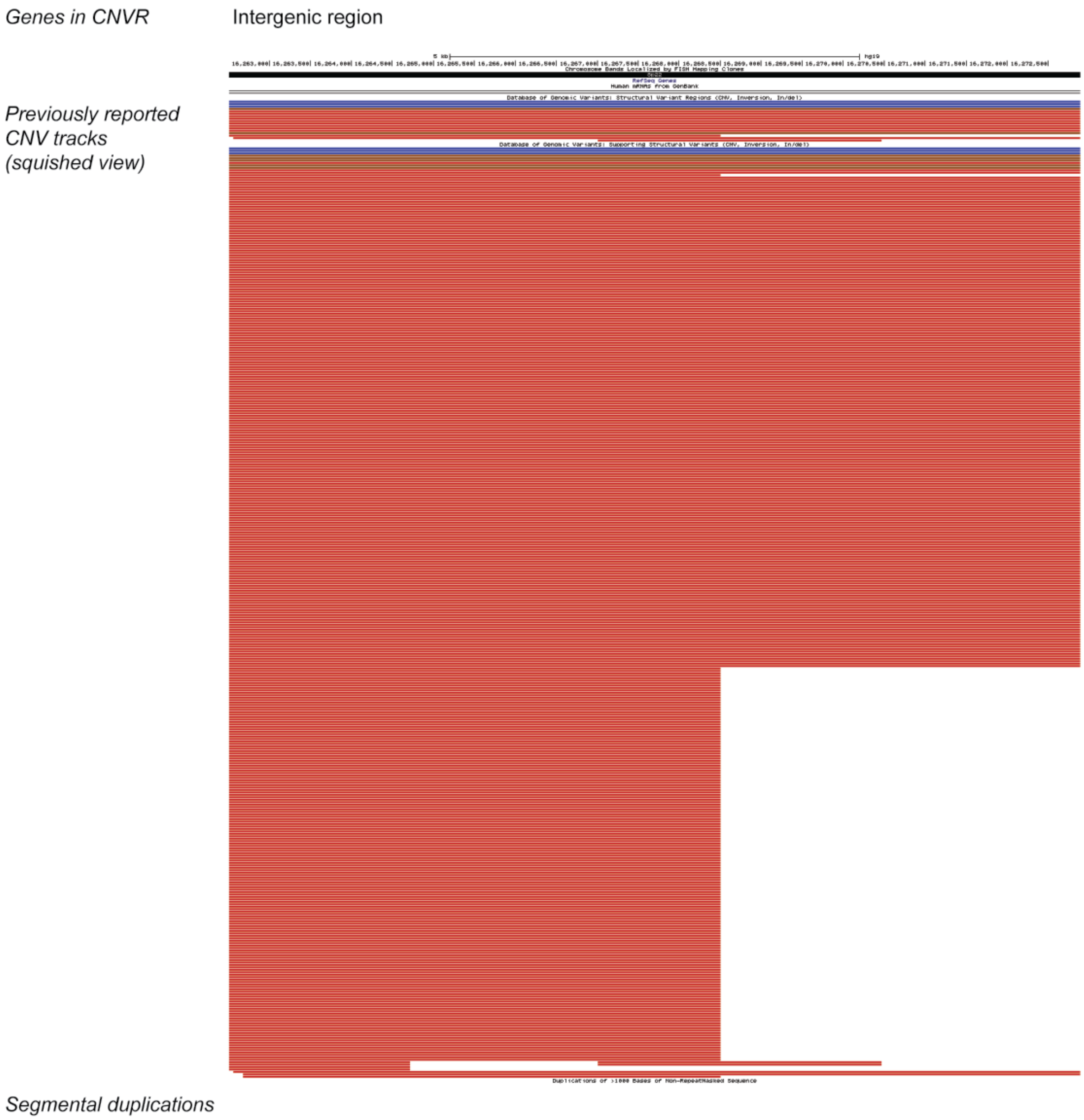

4. Chr12:6243909-6247564, deletion of 3655 bp, intergenic, 10 kb upstream from *VWF* gene.

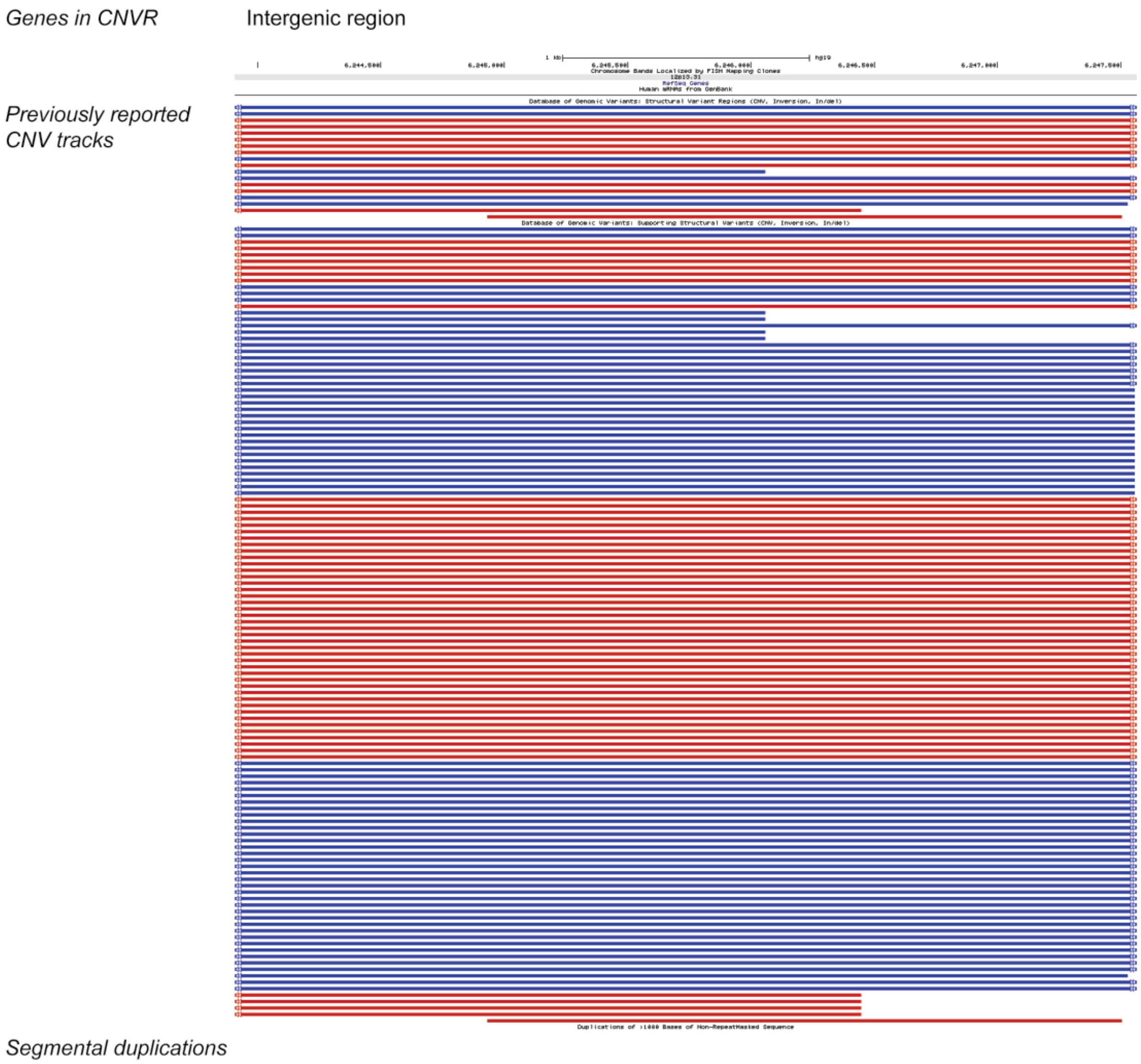

5. Chr14:106028569-106112716, deletion of 84147 bp, overlapping *IGHE* gene.

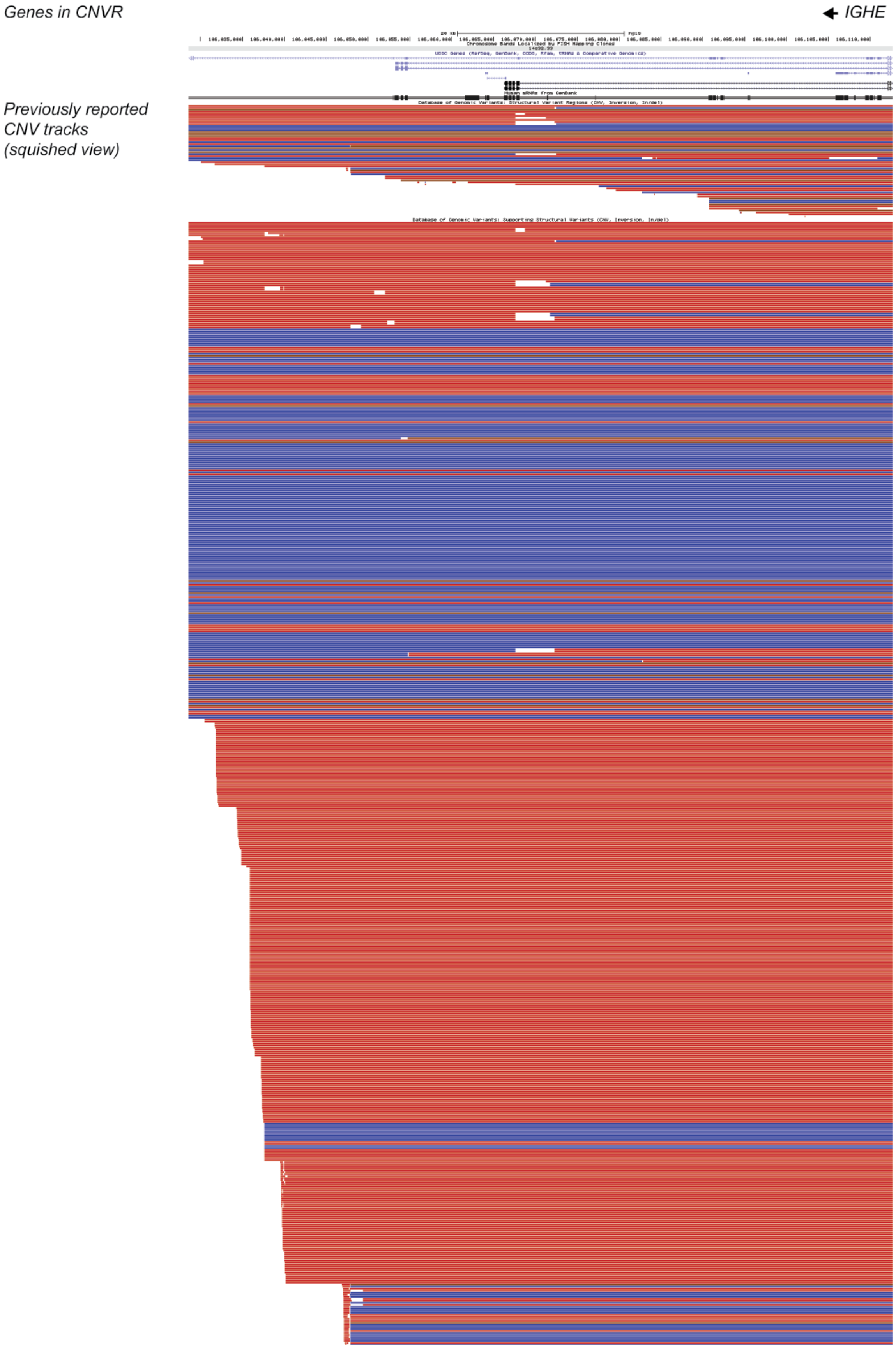

Continued on next page

Previously reported CNV tracks (squished view) Continued

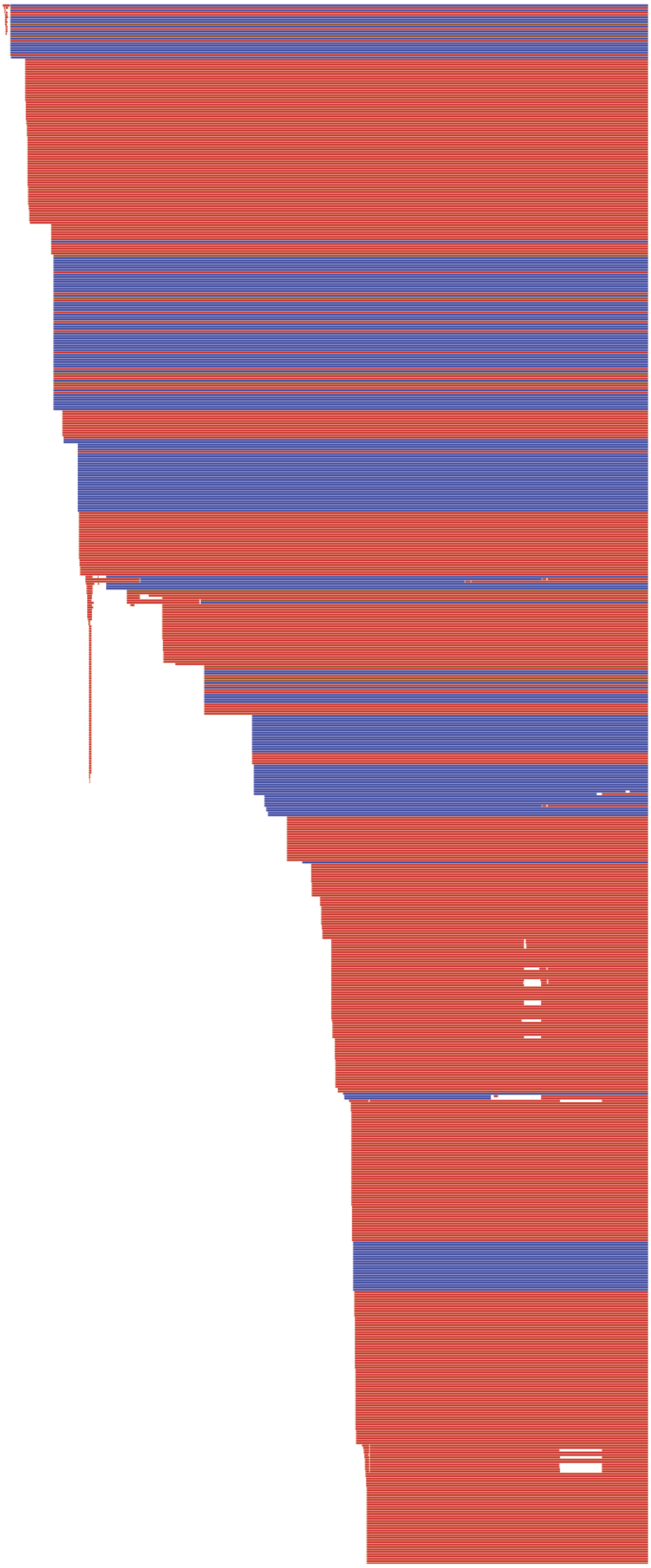

Continued on next page

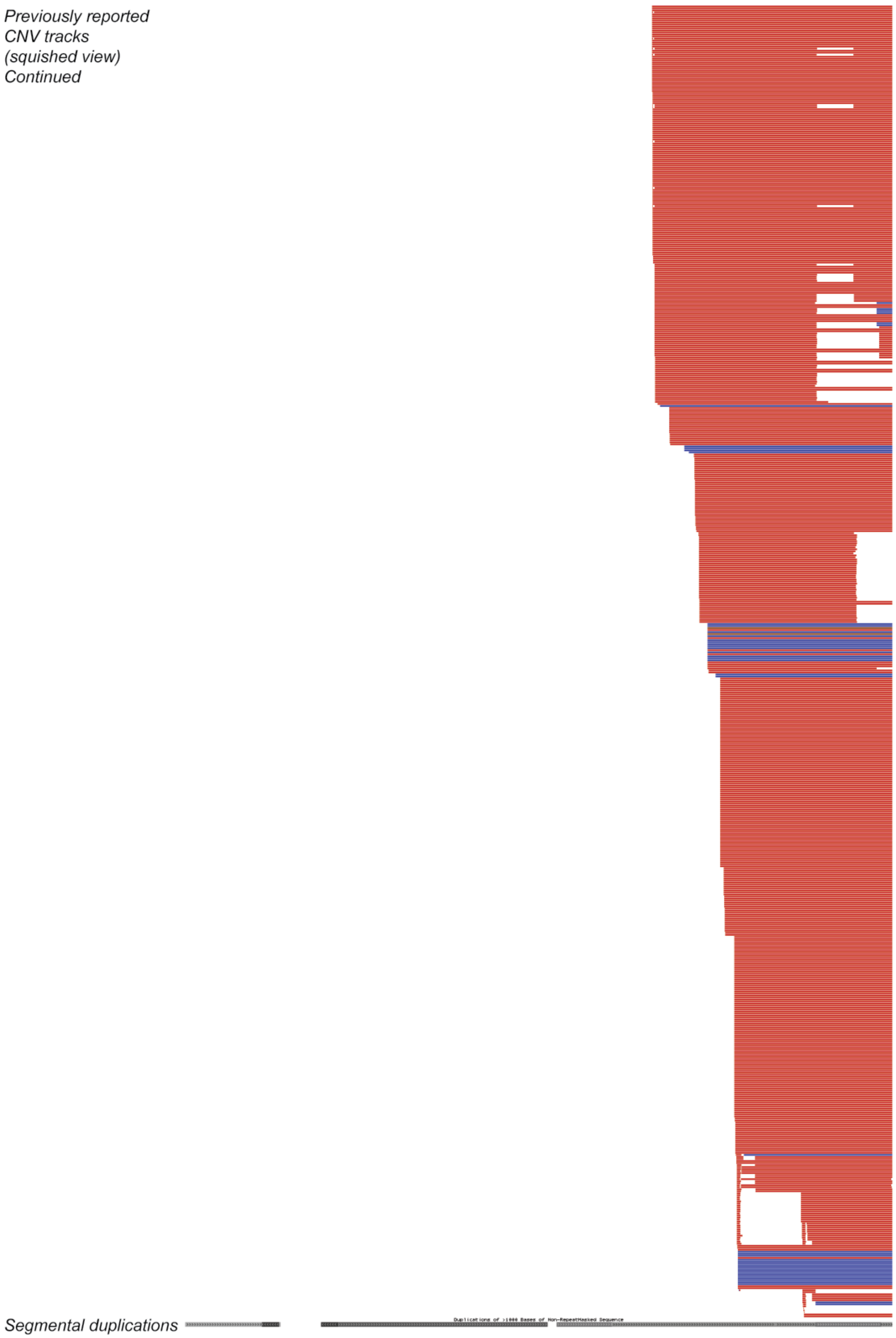
Previously reported
CNV tracks
(squished view)
Continued
Segmental duplications
Duplications of >1000 Bases of Non-RepeatMasked Sequence

Supp. Figure S7. Genomic context flanking the 5p13.3 duplication endpoints in the *PDZD2* and *GOLPH3* genes. Repetitive DNA elements in the DNA sequence 1000 bp up- and downstream from the duplication endpoints in the *PDZD2* (A) and *GOLPH3* gene (B) were identified using RepeatMasker software and are highlighted by lowercase on the grey background with DNA element class given at the end. The position of the 1-nucleotide microhomology at the duplication junction is indicated in red. Genomic positions are given according to hg19.

A. Genomic context of the duplication breakpoint in the *PDZD2* gene (DNA sequence chr5:32105203-32107203).

```
Gcactccagcctgggtgacagagggagaccttgtctcaaaaaaaaacaatcaatcaatcaatcaatcaatccaga
atatatgaagaactatataattcagtaagaaaaatacaagagagaaagtttgggtaaagaaagcaatagcaaatg
gacagaagttaaaacctaggtggccaagaaacacaggaagatgctcaaacttaaatgtattggatgaaaattaga
aatattataaatcattctttaatctatcaagttggccaaaaataaaaaatctcatactatgatgtgttggcaag
gatgtggagaaaggaaaacacatttttttctcgtgcaagtatctactggttaatctttaaggaaaattttagca
gtgcttacgaaaatggaagctatgaaacaatttaaatttctatctgtagcagaataaaatatattcatctaaagg
aaagctaaatagcagttgaaaaaataaattacacctctctctatatactaactgggaccggctcaaaaacagtgt
tgaatgaaaaaaagccaaatgcagattgtgcactgtgtgacaccaatt(L1ME3Cz)CATGGTAAGCATAAGGAa
cctaagaatgtgttcaacaaaagctatgcaagacctttttgggaaaaagcataaaagctttctaaaggacaattt
taaaaaatctatgagtaaatggagagagagacatatcctgctca(L1M5)Aatctgttaagattcagtttggcct
catgtaataggaaatccaaaatagcagtggtttaaacgagagaatttatttctctcttgtgtaaaaagtccaaaa
gctggca(MLT1J)AACCTCTGCTTTGTACAACACTGAATGTTTCCTAAAGGagcagtccccaagctttttggca
ccagggactggttttgtggaagacaatttttccatggtgagggtttgggtggggatggtttcaggatgattcag
gtgcattacatttattgtatactttatttctgttattacattttaat/a/Breakpoint(Chr5:32106204)
tataatgaaataattatacaactcaccatcacgtagaatctgtgggagccctgagcttgttttcttgcaaccagg
catcccatctgggggtgatgggagacaatgacagatcatcaggcattagattctcataaggaacatgcaacctag
atccctcgcatatgcagttcacaatagggttcacactcacgtgagaatctaatgctgctgctgatctgacaggag
gcagagctcaggtggtagtgtgagtgacggtgagaggctgtgaacagaggaagctttgctcactcacccactgct
cacctgctgtgcagccctcctgccaccagtctgtggcccaagggttgaggacccctg(MER1A)TCCTAGAAGAC
TTTCCTCtacttcatctcattcagtccttctaacaacctagtaagataacaagacagatatcatcatcaccattt
tgcagatgagaaaactggagtttggagaaatctgtaacttgcctgaagtcaca(MIRc)GATTTGAGACCAAACC
CAGTCTGATTGCTCCTTCAGTGTGCGTCAGGCTGTTCTGGTTGCCTGGAACCTCCCATGTCCTGAAGCCCAGGTG
AACACTCACAAAATTCTTTTGTTTCACATAACCTGAAGGCCTTCGGTTAATATGCTAGAATAGGACATTGGGTAC
TAAGTTAACACAAATATAATTGGTTCTTTGGTCCTTCCAAAAATGTATCTATCTCAAGAAAAGCAAGGACTAGGG
ACCAAATCATTTTCCTGGGGCCCACTACAGGGCTGATATTGGTGAGGTCTCTCTTCTAATGTAACTAACTGCCTC
TATCATTTACCACAGAAAATGTTTTTGTCCTTGTGTGGGCTGGATTTCTTCCAAGCTGTATTTTTATCACATAAT
ATTTATTTCAATAATTTCAATAACGACTTTCAGTAATTTAGGATGAAATCGATACATCTGCTCAGTCTTTTGAGG
TCTAATCAAAATTTCTGAGAAAATTTTTTTCACACTTG
```

B. Genomic context of the duplication breakpoint in the *GOLPH3* gene (DNA sequence chr5:32166776-32168776).

```
tggtggcaggcgcctgtaatcctagctacttgggaggctgaggcaggagaatcacttgaacccaggaggcggagt
tgcagtgggccaagacggtgccattgcacttcagcccgagtgataaaagcaagactccatcttaaaaaaaaaaaa
aaa(AluSx)GCAGGAGGAGGACAAGCACTGTCTTTTTGTGCTGCTTCAAAGGGAGACTCACAACATGAAGATAA
ATCCTCTAAACAAAGACTACAGCATAGTTTTATACTATTTAATCTGATTAATGGCAGTAGAAAAGTTATGCTGTT
AGAAGGGGGGGGGAGTTTGCAAAGACATAAAAAAGTGTACAGCCAAAAGCTTAACATTATGTACCAGAACAAGCA
TAATGCCTGATGCCTCCAGGGTATTTAAGATTTACATGTAAGATGATATTATAAAAGTGTATTTCTTTAAATATC
AAAAAAATCCCCCAAATATTTCAAAATCTATCAAAATAACTTCAAAATACAAttcttttgttttgttttgtcttt
tgagatggagttccactcttgtcgcccaggctggagtgcagtggcgcaatcttggctcactgcaacctccgcctc
tcaggttcaagtgattctcctgcctcagcctcccgagtagctgggattacaggtgctcgccaccatgtctggcta
atttttatatttttaatagagacaggggttcaccatgttggccaggcttgtcttgaactcctgacctcaggtgat
ccatccacctcggcttcccaaagtactgggattacaggtgtgagccaccgcacccagcc(AluSx)TTCAAAATA
```

CAATTCTAAAACGTAATTCAAATGCTCCATACAAGAACTCTCAGGTAGTCCAAAATTAACAAATATCAAATATTA
GTATAACTTATGAGATGAGTTAGTGAATATTTTGACCATATTGTAAAAGAATCCCAAAGCACTAAAAAGTAATTT
CAACAGTATAAAATCTTGTTGAAAAGGTATAATAAGCAg**/a/Breakpoint(Chr5:32167776)**ccaggcgt
ggtggctcacacctgtaatcccagaactttgggaggccaaggcgggaagattgcttgaggccaggagttcgagac
taggcaacatagtgagaccacgtctctacaaaaaaaaattttttttttaattagctgggcatgatagggtgcgc
ctatagtcccagctattcaggagactgaggagggaggattgcttgagttcaggaggtcgaggttgcaatgagcca
tgatcacaccaccacactccagtctggaagacaaagctaggccctgtctcaaaagaaaaaaa**(AluJb)**GTACAA
TCAAATAGTTTAATGAACTTCAAAAGTAATTTCTGAGATCCTGTATATCTCATACCTGTGTTTAAACACCTGAAA
AAAATAGATAAAAATTCAAAACTTCAAAAAATATGAAGGAAATATCATTTGTCTTAGAAACAACGAAAATTCATT
AAAAACTCAGAAGCCTTTATTATATTTTAAATTACTAAAACTCAGCACATCTGGAAATTCTGAATACTTGTTGAC
TGAATTTCTGATTTACAGATTTGATATGTAGAGATGGTGGACATCACCACACGTATAGAAAGGAAAACAATTTTT
GTTAAGTTATACATCTTCATCACTATTATACTTCAAAGTAGTGACCTTGGGAACCTTATTCCAAAAATGGAAAAC
ATCTTTGGAGCCTGTGTAAAAGCCACACAAAAGTCAGTTCTATCATTTTGGTTAATCTTAAGTATTTTACCCCAT
TACATATTATTTACCTCAAGTACCTACTTATTTTTCAAGACATAGAGTTAAGTTACTGTATTACAGTTCTTGTCT
ACAAGCATTAGAAACATTAGTAACTGACTCTGGCTATCTTTAAAAAGCAACTTTACTGGAAGGCTGTTGAAACTC
ACAACACTGAAAGATGACTGGAAAATCAGACTGGAAAAAAACAGTGCCAACAGACCAGAGAAAGCAGCTCAAAGA
ACCATGCCAAAGAACAGTCTGATC

SUPP. TABLES

Supp. Table S1. Characteristics of study subjects

	All subjects	Female subjects		Male subjects	
Study population	No of samples	No of samples	Median age at event, years (5–95%)	No of samples	Median age at event, years (5–95%)
Recurrent miscarriage cases[a]					
All	558	309		249	
Estonia	119	80	30.0 (21.0-37.0)	39	29.0 (23.0-38.3)
Denmark	439	229	30.0 (23.0-38.0)	210	32.0 (24.0-40.6)
Fertile controls[b]					
All	205	205		n.a.	
Estonia	90	90	31.0 (22.9-38.5)	n.a.	
Denmark	115	115	30.0 (25.0-34.4)	n.a.	
Population-based cohort from Estonian Biobank (EGCUT)[c]					
Estonia	1000	496	44.0 (21.0-72.0)	504	42.0 (19-74)

[a]RM patients have experienced ≥3 consecutive miscarriages before gestational week 22. The age at event represents the age at the time of the 3rd miscarriage.
[b]Fertile control women have given birth to at least two (Denmark) or three (Estonian) children and have experienced no miscarriages. The age at event represents the age at the time of the 2nd (Denmark) or 3rd (Estonia) birth.
[c]Estonian population-based cohort sample drawn from the Estonian Biobank of the Estonian Genome Center at the University of Tartu (EGCUT). The age at event represents the age at the time of recruitment.
n.a., not applicable.

Supp. Table S2. Characteristics of Estonian study subjects analyzed in the discovery phase (n = 70)

Study population	No of samples	Median age at event, years (5–95%)[a]
RM cases[b]	43	29.0 (21.0-38.0)
Fertile controls[c]	27	30.0 (24.0-34.7)

[a]Age at the time of the 3rd miscarriage for patients or 3rd birth for fertile controls.
[b]RM patients represent either female (n = 27) or male (n = 16) partners of RM couples, who have experienced ≥3 consecutive miscarriages before gestational week 22.
[c]Fertile control women have given birth to at least three children and have experienced no miscarriages.

Supp. Table S3. Summary of copy number variable regions identified based on Illumina SNP-array genotyping and CNV calling in Estonian discovery phase sample set (n = 70). Genomic coordinates are given according to hg18.

A. CNV regions in RM cases

CNVR ID	Chr	CNVR Start pos	CNVR End pos	Chr band	CNVR length	CNV freq in cases	DEL freq in cases	DUPL freq in cases
RM_CNVR_1	1	1125105	1680910	1p36.33	555805	5/43	5	0
RM_CNVR_2	1	2351880	2434274	1p36.32	82394	2/43	2	0
RM_CNVR_3	1	2690232	3189077	1p36.32	498845	1/43	0	1
RM_CNVR_4	1	6223869	6452776	1p36.31	228907	1/43	1	0
RM_CNVR_5	1	9259704	9323455	1p36.22	63751	1/43	0	1
RM_CNVR_6	1	10623035	10665950	1p36.22	42915	1/43	1	0
RM_CNVR_7	1	40785602	40799336	1p34.2	13734	1/43	1	0
RM_CNVR_8	1	45202805	45227872	1p34.1	25067	1/43	0	1
RM_CNVR_9	1	49701076	49751329	1p33	50253	1/43	1	0
RM_CNVR_10	1	102434268	102571645	1p21.1	137377	1/43	1	0
RM_CNVR_11	1	147306554	147414362	1q21.1	107808	1/43	1	0
RM_CNVR_12	1	147435422	147983660	1q21.1	548238	1/43	1	0
RM_CNVR_13	1	148875518	149203953	1q21.2	328435	1/43	0	1
RM_CNVR_14	1	151025143	151033105	1q21.3	7962	1/43	1	0
RM_CNVR_15	1	156760350	156764196	1q23.1	3846	1/43	1	0
RM_CNVR_16	1	189824645	189942040	1q31.2	117395	1/43	0	1
RM_CNVR_17	1	195089923	195163711	1q31.3	73788	4/43	2	2
RM_CNVR_18	1	200684186	200797989	1q32.1	113803	1/43	0	1
RM_CNVR_19	1	220564149	220600036	1q41	35887	1/43	1	0
RM_CNVR_20	1	224441890	224555449	1q42.12	113559	2/43	2	0
RM_CNVR_21	1	226576050	226754571	1q42.13	178521	1/43	1	0
RM_CNVR_22	1	229778112	229879757	1q42.2	101645	1/43	0	1
RM_CNVR_23	1	237159862	237190804	1q43	30942	1/43	1	0
RM_CNVR_24	1	239936490	239953804	1q43	17314	1/43	1	0
RM_CNVR_25	1	243226190	243245811	1q44	19621	1/43	0	1
RM_CNVR_26	1	246837884	246852068	1q44	14184	2/43	2	0
RM_CNVR_27	2	79910	190354	2p25.3	110444	1/43	0	1
RM_CNVR_28	2	4191253	4199818	2p25.3	8565	5/43	5	0
RM_CNVR_29	2	14273172	14285501	2p24.3	12329	1/43	0	1
RM_CNVR_30	2	17086609	17095859	2p24.2	9250	1/43	1	0
RM_CNVR_31	2	28016365	28021919	2p23.2	5554	1/43	0	1
RM_CNVR_32	2	34556561	34580068	2p22.3	23507	5/43	2	3

CNVR ID	Chr	CNVR Start pos	CNVR End pos	Chr band	CNVR length	CNV freq in cases	DEL freq in cases	DUPL freq in cases
RM_CNVR_33	2	41094996	41101972	2p22.1	6976	1/43	1	0
RM_CNVR_34	2	51776071	51781546	2p16.3	5475	8/43	8	0
RM_CNVR_35	2	52613957	52637176	2p16.3	23219	4/43	3	1
RM_CNVR_36	2	57256144	57319307	2p16.1	63163	2/43	0	2
RM_CNVR_37	2	65643545	65647808	2p14	4263	1/43	0	1
RM_CNVR_38	2	89568953	89948524	2p11.2	379571	2/43	2	0
RM_CNVR_39	2	103847757	103872210	2q12.1	24453	1/43	0	1
RM_CNVR_40	2	110085004	111135899	2q13	1050895	2/43	1	1
RM_CNVR_41	2	116624720	116666628	2q14.1	41908	1/43	0	1
RM_CNVR_42	2	116692536	116789226	2q14.1	96690	1/43	0	1
RM_CNVR_43	2	133976082	134028183	2q21.2	52101	1/43	1	0
RM_CNVR_44	2	135978882	135983885	2q21.3	5003	1/43	1	0
RM_CNVR_45	2	136294664	136307216	2q21.3	12552	1/43	0	1
RM_CNVR_46	2	140546178	140608609	2q22.1	62431	1/43	1	0
RM_CNVR_47	2	141842203	141867875	2q22.1	25672	1/43	0	1
RM_CNVR_48	2	146583025	146592386	2q22.3	9361	1/43	1	0
RM_CNVR_49	2	153026585	153057411	2q23.3	30826	1/43	0	1
RM_CNVR_50	2	161037890	161140312	2q24.2	102422	1/43	1	0
RM_CNVR_51	2	180123158	180129913	2q31.2	6755	1/43	1	0
RM_CNVR_52	2	184395055	184402559	2q32.1	7504	1/43	1	0
RM_CNVR_53	2	188169683	188507005	2q32.1	337322	1/43	1	0
RM_CNVR_54	2	195017393	195496752	2q32.3	479359	1/43	1	0
RM_CNVR_55	2	195639537	195689951	2q32.3	50414	1/43	1	0
RM_CNVR_56	2	230696392	230703691	2q36.3-2q37.1	7299	1/43	0	1
RM_CNVR_57	2	241204664	241332581	2q37.3	127917	1/43	1	0
RM_CNVR_58	2	242151629	242692820	2q37.3	541191	2/43	2	0
RM_CNVR_59	3	41894	103697	3p26.3	61803	2/43	0	2
RM_CNVR_60	3	1561672	1697181	3p26.3	135509	1/43	0	1
RM_CNVR_61	3	8801023	8832963	3p25.3	31940	1/43	0	1
RM_CNVR_62	3	37954886	37957761	3p22.2	2875	2/43	2	0
RM_CNVR_63	3	65166887	65187636	3p14.1	20749	2/43	2	0
RM_CNVR_64	3	75502426	75719139	3p12.3	216713	1/43	1	0
RM_CNVR_65	3	82823888	83352420	3p12.2	528532	1/43	1	0
RM_CNVR_66	3	89489946	89499861	3p11.1	9915	1/43	1	0
RM_CNVR_67	3	101840361	101916282	3q12.2	75921	1/43	0	1
RM_CNVR_68	3	116143746	116150586	3q13.31	6840	1/43	1	0
RM_CNVR_69	3	130286930	130389647	3q21.3	102717	1/43	1	0
RM_CNVR_70	3	152997280	153028731	3q25.1	31451	2/43	2	0

CNVR ID	Chr	CNVR Start pos	CNVR End pos	Chr band	CNVR length	CNV freq in cases	DEL freq in cases	DUPL freq in cases
RM_CNVR_71	3	163603327	163625169	3q26.1	21842	3/43	3	0
RM_CNVR_72	3	164004033	164101579	3q26.1	97546	7/43	5	2
RM_CNVR_73	3	166524485	166560107	3q26.1	35622	2/43	2	0
RM_CNVR_74	3	167766851	167902738	3q26.1	135887	1/43	1	0
RM_CNVR_75	3	168010845	168016121	3q26.1	5276	1/43	0	1
RM_CNVR_76	3	176564661	176569238	3q26.31	4577	4/43	4	0
RM_CNVR_77	3	177371924	177385694	3q26.32	13770	1/43	1	0
RM_CNVR_78	3	190847118	190849457	3q28	2339	1/43	1	0
RM_CNVR_79	3	191220845	191221392	3q28	547	2/43	1	1
RM_CNVR_80	4	4375134	5074013	4p16.2	698879	1/43	0	1
RM_CNVR_81	4	9032348	9079320	4p16.1	46972	1/43	0	1
RM_CNVR_82	4	22504558	22527192	4p15.31	22634	1/43	1	0
RM_CNVR_83	4	57759221	57793311	4q12	34090	1/43	0	1
RM_CNVR_84	4	63352170	63357704	4q13.1	5534	5/43	5	0
RM_CNVR_85	4	64380191	64389207	4q13.1	9016	5/43	5	0
RM_CNVR_86	4	69097539	69163188	4q13.2	65649	5/43	3	2
RM_CNVR_87	4	70216715	70236428	4q13.2	19713	1/43	1	0
RM_CNVR_88	4	85739667	85764668	4q21.23	25001	1/43	0	1
RM_CNVR_89	4	87195437	87198277	4q21.3	2840	2/43	2	0
RM_CNVR_90	4	94659049	94848390	4q22.2	189341	1/43	1	0
RM_CNVR_91	4	104973072	104980491	4q24	7419	1/43	1	0
RM_CNVR_92	4	108285187	108292659	4q25	7472	2/43	2	0
RM_CNVR_93	4	115398433	115401739	4q26	3306	4/43	2	2
RM_CNVR_94	4	138403297	138406802	4q28.3	3505	1/43	1	0
RM_CNVR_95	4	153514325	153719440	4q31.3	205115	1/43	0	1
RM_CNVR_96	4	158781719	158783730	4q32.1	2011	1/43	1	0
RM_CNVR_97	4	161271979	161291569	4q32.1	19590	1/43	1	0
RM_CNVR_98	4	162172873	162223074	4q32.2	50201	1/43	0	1
RM_CNVR_99	4	186472542	186505570	4q35.1	33028	1/43	1	0
RM_CNVR_100	4	187220336	187281155	4q35.1	60819	1/43	0	1
RM_CNVR_101	4	188103337	188108448	4q35.2	5111	1/43	0	1
RM_CNVR_102	5	8756085	8797557	5p15.2	41472	3/43	2	1
RM_CNVR_103	5	9955340	9976541	5p15.2	21201	2/43	2	0
RM_CNVR_104	5	19984687	20274531	5p14.3	289844	1/43	1	0
RM_CNVR_105	5	32142841	32202977	5p13.3	60136	2/43	0	2
RM_CNVR_106	5	97074222	97127572	5q15	53350	4/43	4	0
RM_CNVR_107	5	104401279	104677904	5q21.2-5q21.3	276625	2/43	2	0
RM_CNVR_108	5	106257423	106258797	5q21.3	1374	1/43	1	0

CNVR ID	Chr	CNVR Start pos	CNVR End pos	Chr band	CNVR length	CNV freq in cases	DEL freq in cases	DUPL freq in cases
RM_CNVR_109	5	111369344	111463668	5q22.1	94324	1/43	1	0
RM_CNVR_110	5	151493576	151499003	5q33.1	5427	4/43	4	0
RM_CNVR_111	5	163429884	163503982	5q34	74098	1/43	1	0
RM_CNVR_112	5	178761460	178993601	5q35.3	232141	1/43	0	1
RM_CNVR_113	6	29959422	30009547	6p21.33	50125	6/43	6	0
RM_CNVR_114	6	31329464	31337835	6p21.33	8371	1/43	1	0
RM_CNVR_115	6	31384589	31412642	6p21.33	28053	6/43	6	0
RM_CNVR_116	6	32072225	32161107	6p21.32	88882	3/43	2	1
RM_CNVR_117	6	32550064	32666924	6p21.32	116860	6/43	4	2
RM_CNVR_118	6	32710460	32731201	6p21.32	20741	1/43	0	1
RM_CNVR_119	6	32740809	32763196	6p21.32	22387	5/43	4	1
RM_CNVR_120	6	32871088	32883787	6p21.32	12699	1/43	0	1
RM_CNVR_121	6	54093298	54113002	6p12.1	19704	1/43	1	0
RM_CNVR_122	6	62241860	62281216	6q11.1	39356	1/43	0	1
RM_CNVR_123	6	65873533	67121600	6q12	1248067	4/43	4	0
RM_CNVR_124	6	75032898	75097166	6q13	64268	1/43	0	1
RM_CNVR_125	6	79029649	79090197	6q14.1	60548	11/43	6	5
RM_CNVR_126	6	89212325	89219718	6q15	7393	1/43	0	1
RM_CNVR_127	6	109094877	109114925	6q21	20048	1/43	0	1
RM_CNVR_128	6	110510873	110521442	6q21	10569	1/43	1	0
RM_CNVR_129	6	131771552	131939901	6q23.2	168349	1/43	0	1
RM_CNVR_130	6	161943593	162413672	6q26	470079	1/43	0	1
RM_CNVR_131	7	1467240	1560184	7p22.3	92944	1/43	1	0
RM_CNVR_132	7	9521207	9543940	7p21.3	22733	1/43	1	0
RM_CNVR_133	7	20442719	20534687	7p15.3	91968	1/43	0	1
RM_CNVR_134	7	55350419	56337310	7p11.2	986891	2/43	1	1
RM_CNVR_135	7	61681059	62354262	7q11.21	673203	5/43	4	1
RM_CNVR_136	7	79193132	79321416	7q21.11	128284	1/43	0	1
RM_CNVR_137	7	83283614	83297844	7q21.11	14230	1/43	1	0
RM_CNVR_138	7	100755083	100914175	7q22.1	159092	1/43	0	1
RM_CNVR_139	7	104405554	104439907	7q22.2	34353	1/43	0	1
RM_CNVR_140	7	110679462	111065681	7q31.1	386219	3/43	3	0
RM_CNVR_141	7	117796542	118009548	7q31.31	213006	1/43	0	1
RM_CNVR_142	7	118901124	118948979	7q31.31	47855	2/43	2	0
RM_CNVR_143	7	120092385	120099982	7q31.31	7597	1/43	1	0
RM_CNVR_144	7	141423761	141439888	7q34	16127	1/43	1	0
RM_CNVR_145	8	584761	588391	8p23.3	3630	3/43	3	0
RM_CNVR_146	8	4463376	4471906	8p23.2	8530	1/43	1	0

CNVR ID	Chr	CNVR Start pos	CNVR End pos	Chr band	CNVR length	CNV freq in cases	DEL freq in cases	DUPL freq in cases
RM_CNVR_147	8	5550690	5572789	8p23.2	22099	1/43	1	0
RM_CNVR_148	8	5586807	5592495	8p23.2	5688	2/43	2	0
RM_CNVR_149	8	7176768	7947780	8p23.1	771012	2/43	2	0
RM_CNVR_150	8	9093182	9100437	8p23.1	7255	1/43	1	0
RM_CNVR_151	8	15821208	15830711	8p22	9503	1/43	1	0
RM_CNVR_152	8	16306880	16317263	8p22	10383	5/43	5	0
RM_CNVR_153	8	39344125	39627026	8p11.23-8p11.22	282901	25/43	17	8
RM_CNVR_154	8	47016137	47406312	8q11.1	390175	1/43	0	1
RM_CNVR_155	8	51288510	51298097	8q11.22	9587	1/43	0	1
RM_CNVR_156	8	85420095	85428552	8q21.2	8457	3/43	3	0
RM_CNVR_157	8	107157602	107159533	8q23.1	1931	1/43	1	0
RM_CNVR_158	8	128144872	128162660	8q24.21	17788	1/43	1	0
RM_CNVR_159	8	142796991	142825904	8q24.3	28913	1/43	1	0
RM_CNVR_160	8	145064091	145358471	8q24.3	294380	2/43	2	0
RM_CNVR_161	8	145549104	145656204	8q24.3	107100	1/43	1	0
RM_CNVR_162	9	385025	702156	9p24.3	317131	1/43	0	1
RM_CNVR_163	9	9727201	9887866	9p23	160665	1/43	1	0
RM_CNVR_164	9	23062095	23064432	9p21.3	2337	1/43	1	0
RM_CNVR_165	9	30276600	30743998	9p21.1	467398	1/43	1	0
RM_CNVR_166	9	43570503	43730292	9p11.2	159789	2/43	2	0
RM_CNVR_167	9	71289871	71308782	9q21.11	18911	1/43	1	0
RM_CNVR_168	9	74792723	75101266	9q21.13	308543	2/43	0	2
RM_CNVR_169	9	80518252	80536668	9q21.31	18416	1/43	1	0
RM_CNVR_170	9	82510013	82513047	9q21.31	3034	1/43	1	0
RM_CNVR_171	9	89322041	89323546	9q21.33	1505	1/43	0	1
RM_CNVR_172	9	104803937	104840064	9q31.1	36127	1/43	1	0
RM_CNVR_173	9	137223172	137244318	9q34.3	21146	1/43	1	0
RM_CNVR_174	9	137720668	137778203	9q34.3	57535	1/43	1	0
RM_CNVR_175	9	138612610	138716241	9q34.3	103631	1/43	1	0
RM_CNVR_176	9	139188163	139421268	9q34.3	233105	3/43	3	0
RM_CNVR_177	10	17688978	17797302	10p12.33	108324	1/43	1	0
RM_CNVR_178	10	41679826	41702521	10q11.1	22695	1/43	1	0
RM_CNVR_179	10	46770968	47519753	10q11.22	748785	4/43	0	4
RM_CNVR_180	10	55576118	55708837	10q21.1	132719	2/43	2	0
RM_CNVR_181	10	56118892	56144916	10q21.1	26024	1/43	0	1
RM_CNVR_182	10	58519859	58649798	10q21.1	129939	1/43	1	0
RM_CNVR_183	10	58880806	58881704	10q21.1	898	1/43	0	1
RM_CNVR_184	10	66980652	66982114	10q21.3	1462	1/43	1	0
RM_CNVR_185	10	81058202	81113902	10q22.3	55700	1/43	1	0
RM_CNVR_186	10	81552017	81907688	10q22.3	355671	1/43	0	1
RM_CNVR_187	10	82869699	82875955	10q23.1	6256	4/43	4	0

CNVR ID	Chr	CNVR Start pos	CNVR End pos	Chr band	CNVR length	CNV freq in cases	DEL freq in cases	DUPL freq in cases
RM_CNVR_188	10	83940520	83943696	10q23.1	3176	1/43	1	0
RM_CNVR_189	10	90816759	90864448	10q23.31	47689	1/43	1	0
RM_CNVR_190	10	96511239	96537453	10q23.33	26214	1/43	1	0
RM_CNVR_191	10	115737776	117550674	10q25.3	1812898	1/43	1	0
RM_CNVR_192	10	134935744	135034116	10q26.3	98372	1/43	1	0
RM_CNVR_193	10	135102337	135227438	10q26.3	125101	5/43	0	5
RM_CNVR_194	11	539119	631191	11p15.5	92072	1/43	1	0
RM_CNVR_195	11	2142911	2176538	11p15.5	33627	1/43	1	0
RM_CNVR_196	11	2345437	2359323	11p15.5	13886	1/43	1	0
RM_CNVR_197	11	4203196	4292124	11p15.4	88928	1/43	1	0
RM_CNVR_198	11	6456885	6489095	11p15.4	32210	1/43	0	1
RM_CNVR_199	11	25074327	25131774	11p14.3	57447	1/43	1	0
RM_CNVR_200	11	38208761	38272160	11p12	63399	1/43	1	0
RM_CNVR_201	11	49685221	49701670	11p11.12	16449	4/43	4	0
RM_CNVR_202	11	50418414	51228612	11p11.12	810198	1/43	0	1
RM_CNVR_203	11	54627834	54791987	11q11	164153	2/43	2	0
RM_CNVR_204	11	55124465	55209499	11q11	85034	7/43	7	0
RM_CNVR_205	11	69414226	69492477	11q13.3	78251	1/43	1	0
RM_CNVR_206	11	84941372	84948993	11q14.1	7621	1/43	1	0
RM_CNVR_207	11	85982050	85984049	11q14.2	1999	2/43	2	0
RM_CNVR_208	11	86550152	86702707	11q14.2	152555	1/43	1	0
RM_CNVR_209	11	92555192	92571877	11q21	16685	1/43	1	0
RM_CNVR_210	11	97579739	97776889	11q22.1	197150	1/43	1	0
RM_CNVR_211	11	107166452	107175438	11q22.3	8986	3/43	0	3
RM_CNVR_212	11	125747907	125751286	11q24.2	3379	1/43	0	1
RM_CNVR_213	11	126704758	126709174	11q24.2	4416	1/43	1	0
RM_CNVR_214	12	5103788	5228596	12p13.32	124808	1/43	0	1
RM_CNVR_215	12	6114170	6117825	12p13.31	3655	3/43	3	0
RM_CNVR_216	12	18821605	18841637	12p12.3	20032	1/43	0	1
RM_CNVR_217	12	30129221	30132410	12p11.22	3189	3/43	3	0
RM_CNVR_218	12	31157554	31298174	12p11.21	140620	6/43	0	6
RM_CNVR_219	12	31915523	31954269	12p11.21	38746	3/43	0	3
RM_CNVR_220	12	33193705	33197122	12p11.21	3417	6/43	4	2
RM_CNVR_221	12	58227690	58243143	12q14.1	15453	1/43	1	0
RM_CNVR_222	12	58365671	58397920	12q14.1	32249	1/43	0	1
RM_CNVR_223	12	60365037	60401432	12q14.1	36395	1/43	0	1
RM_CNVR_224	12	76878115	76885128	12q21.2	7013	1/43	1	0
RM_CNVR_225	12	128374258	129022189	12q24.32-12q24.33	647931	1/43	0	1
RM_CNVR_226	12	132240072	132288869	12q24.33	48797	1/43	1	0
RM_CNVR_227	13	17941558	18275679	13q11	334121	1/43	0	1
RM_CNVR_228	13	19318175	19338020	13q12.11	19845	1/43	0	1

**CNVR ID**	**Chr**	**CNVR Start pos**	**CNVR End pos**	**Chr band**	**CNVR length**	**CNV freq in cases**	**DEL freq in cases**	**DUPL freq in cases**
RM_CNVR_229	13	21437167	21442788	13q12.11	5621	1/43	0	1
RM_CNVR_230	13	27410585	27412987	13q12.2	2402	1/43	0	1
RM_CNVR_231	13	35670381	35671944	13q13.3	1563	1/43	0	1
RM_CNVR_232	13	63201537	63277373	13q21.31	75836	1/43	1	0
RM_CNVR_233	13	63310840	63415259	13q21.31	104419	1/43	1	0
RM_CNVR_234	13	68150996	68161739	13q21.33	10743	4/43	4	0
RM_CNVR_235	13	68444718	68673098	13q21.33	228380	1/43	1	0
RM_CNVR_236	13	70396781	70408794	13q21.33	12013	1/43	0	1
RM_CNVR_237	13	71023642	71246769	13q21.33	223127	1/43	1	0
RM_CNVR_238	13	83001708	83055928	13q31.1	54220	1/43	1	0
RM_CNVR_239	13	88613376	88636971	13q31.2	23595	3/43	3	0
RM_CNVR_240	13	94740493	94779857	13q32.1	39364	1/43	0	1
RM_CNVR_241	14	18770048	19494891	14q11.1-14q11.2	724843	3/43	0	3
RM_CNVR_242	14	40679974	40739852	14q21.1	59878	3/43	3	0
RM_CNVR_243	14	43634761	43676255	14q21.3	41494	1/43	1	0
RM_CNVR_244	14	45869740	46414182	14q21.3	544442	1/43	1	0
RM_CNVR_245	14	46744261	46747936	14q21.3	3675	1/43	1	0
RM_CNVR_246	14	66173332	66696347	14q23.3	523015	1/43	1	0
RM_CNVR_247	14	87477641	87489111	14q31.3	11470	1/43	1	0
RM_CNVR_248	14	90960430	91014664	14q32.12	54234	1/43	0	1
RM_CNVR_249	14	103910861	104008599	14q32.33	97738	1/43	0	1
RM_CNVR_250	14	104149296	104419597	14q32.33	270301	1/43	1	0
RM_CNVR_251	14	104623128	106058612	14q32.33	1435484	4/43	3	1
RM_CNVR_252	14	106230905	106256042	14q32.33	25137	1/43	0	1
RM_CNVR_253	15	19157192	20009688	15q11.2	852496	6/43	4	2
RM_CNVR_254	15	22965530	22985368	15q11.2	19838	1/43	1	0
RM_CNVR_255	15	28398394	28524636	15q13.2	126242	1/43	1	0
RM_CNVR_256	15	30298847	30620951	15q13.3	322104	1/43	1	0
RM_CNVR_257	15	32518050	32595161	15q14	77111	3/43	3	0
RM_CNVR_258	15	49815069	49836002	15q21.2	20933	1/43	0	1
RM_CNVR_259	15	54579894	54582930	15q21.3	3036	2/43	2	0
RM_CNVR_260	15	74650078	74963213	15q24.3	313135	1/43	1	0
RM_CNVR_261	15	85631534	85671028	15q25.3	39494	1/43	1	0
RM_CNVR_262	15	95575552	95650142	15q26.2	74590	5/43	5	0
RM_CNVR_263	16	1132214	1138939	16p13.3	6725	1/43	1	0
RM_CNVR_264	16	1315264	1355332	16p13.3	40068	1/43	0	1
RM_CNVR_265	16	1421021	1455163	16p13.3	34142	1/43	1	0
RM_CNVR_266	16	3647748	3670579	16p13.3	22831	1/43	1	0
RM_CNVR_267	16	15455811	18175011	16p13.11-16p12.3	2719200	2/43	0	2
RM_CNVR_268	16	19858002	19862969	16p12.3	4967	3/43	3	0

CNVR ID	Chr	CNVR Start pos	CNVR End pos	Chr band	CNVR length	CNV freq in cases	DEL freq in cases	DUPL freq in cases
RM_CNVR_269	16	21506626	21746841	16p12.2-16p12.1	240215	1/43	1	0
RM_CNVR_270	16	32419415	32883026	16p11.2	463611	2/43	2	0
RM_CNVR_271	16	68589459	68698988	16q22.1	109529	1/43	0	1
RM_CNVR_272	16	69031435	69115540	16q22.1	84105	1/43	0	1
RM_CNVR_273	16	74096937	74129424	16q23.1	32487	1/43	0	1
RM_CNVR_274	16	75472997	75570738	16q23.1	97741	1/43	1	0
RM_CNVR_275	16	79796319	79861260	16q23.2	64941	1/43	1	0
RM_CNVR_276	16	83366401	83727618	16q24.1	361217	1/43	1	0
RM_CNVR_277	17	41636474	42092850	17q21.31-17q21.32	456376	4/43	1	3
RM_CNVR_278	17	47817225	47861925	17q22	44700	1/43	0	1
RM_CNVR_279	17	48306578	48670836	17q22	364258	1/43	1	0
RM_CNVR_280	17	51319462	51338491	17q22	19029	1/43	1	0
RM_CNVR_281	18	36514913	36533818	18q12.3	18905	8/43	8	0
RM_CNVR_282	18	37985206	38054891	18q12.3	69685	1/43	1	0
RM_CNVR_283	18	64773253	64851232	18q22.1	77979	1/43	1	0
RM_CNVR_284	18	64897188	64906488	18q22.1-18q22.2	9300	3/43	3	0
RM_CNVR_285	19	20404485	20507068	19p12	102583	1/43	1	0
RM_CNVR_286	19	20625937	20772798	19p12	146861	1/43	0	1
RM_CNVR_287	19	32629673	32679064	19q12	49391	1/43	0	1
RM_CNVR_288	19	48009908	48370536	19q13.31	360628	1/43	1	0
RM_CNVR_289	19	58206416	58240762	19q13.41	34346	1/43	0	1
RM_CNVR_290	19	58635650	58705990	19q13.41	70340	1/43	1	0
RM_CNVR_291	19	59941382	60069820	19q13.42	128438	4/43	1	3
RM_CNVR_292	20	28059305	28121437	20q11.1	62132	1/43	1	0
RM_CNVR_293	20	52081775	52088118	20q13.2	6343	1/43	1	0
RM_CNVR_294	20	54842260	54855185	20q13.31	12925	1/43	1	0
RM_CNVR_295	20	55879430	55902010	20q13.31-20q13.32	22580	1/43	1	0
RM_CNVR_296	20	60230953	60501042	20q13.33	270089	3/43	3	0
RM_CNVR_297	20	61323074	61350614	20q13.33	27540	1/43	1	0
RM_CNVR_298	21	9730102	9849404	21p11.2	119302	1/43	0	1
RM_CNVR_299	21	13323935	14197852	21q11.2	873917	2/43	1	1
RM_CNVR_300	21	15882461	16035019	21q21.1	152558	1/43	0	1
RM_CNVR_301	21	16322787	16381378	21q21.1	58591	1/43	1	0
RM_CNVR_302	21	16589591	16615822	21q21.1	26231	1/43	1	0
RM_CNVR_303	21	17632242	17666286	21q21.1	34044	1/43	0	1
RM_CNVR_304	21	18981497	19000295	21q21.1	18798	1/43	0	1
RM_CNVR_305	21	20227640	20261725	21q21.1	34085	1/43	1	0
RM_CNVR_306	21	31008194	31164645	21q22.11	156451	1/43	0	1
RM_CNVR_307	21	43958296	44082475	21q22.3	124179	1/43	0	1
RM_CNVR_308	22	14494244	14629090	22q11.1	134846	1/43	0	1

CNVR ID	Chr	CNVR Start pos	CNVR End pos	Chr band	CNVR length	CNV freq in cases	DEL freq in cases	DUPL freq in cases
RM_CNVR_309	22	17281004	17406613	22q11.21	125609	1/43	0	1
RM_CNVR_310	22	20679088	20897762	22q11.22	218674	1/43	0	1
RM_CNVR_311	22	23999569	24244593	22q11.23	245024	2/43	1	1
RM_CNVR_312	22	37559539	37630882	22q13.1	71343	1/43	1	0
RM_CNVR_313	22	47038544	47109577	22q13.32	71033	1/43	0	1
RM_CNVR_314	X	15189545	15192743	Xp22.2	3198	1/43	0	1
RM_CNVR_315	X	27000081	27234211	Xp21.3	234130	1/43	0	1
RM_CNVR_316	X	45080594	45189474	Xp11.3	108880	1/43	0	1
RM_CNVR_317	X	47796546	47851087	Xp11.23	54541	1/43	1	0
RM_CNVR_318	X	58265605	58580762	Xp11.1	315157	1/43	0	1
RM_CNVR_319	X	61599263	61808165	Xq11.1	208902	3/43	0	3
RM_CNVR_320	X	64940132	64955287	Xq11.1	15155	1/43	1	0
RM_CNVR_321	X	73480776	73552592	Xq13.2	71816	1/43	0	1
RM_CNVR_322	X	89185625	89750321	Xq21.31	564696	1/43	0	1
RM_CNVR_323	X	111426298	111923923	Xq23	497625	1/43	0	1
RM_CNVR_324	X	115212232	115278016	Xq23	65784	1/43	0	1
RM_CNVR_325	X	134583959	134738060	Xq26.3	154101	1/43	0	1
RM_CNVR_326	X	146208170	146259262	Xq27.3	51092	1/43	0	1
RM_CNVR_327	X	153482887	153622054	Xq28	139167	1/43	0	1

B. CNV regions in fertile controls

CNVR ID	Chr	CNVR Start pos	CNVR End pos	Chr band	CNVR length	CNV freq in cases	DEL freq in cases	DUPL freq in cases
FFC_CNVR_1	1	72821273	72855127	1p31.1	33854	1/27	1	0
FFC_CNVR_2	1	103388526	103443062	1p21.1	54536	1/27	0	1
FFC_CNVR_3	1	111182243	111189062	1p13.3	6819	1/27	1	0
FFC_CNVR_4	1	120870371	121180077	1p11.2-1p11.1	309706	1/27	0	1
FFC_CNVR_5	1	146174007	146325557	1q21.1	151550	3/27	0	3
FFC_CNVR_6	1	164052127	164107035	1q24.1	54908	1/27	1	0
FFC_CNVR_7	1	195037748	195148967	1q31.3	111219	2/27	2	0
FFC_CNVR_8	1	214521106	214542165	1q41	21059	1/27	1	0
FFC_CNVR_9	1	214696039	214723882	1q41	27843	1/27	1	0
FFC_CNVR_10	1	225362339	225488857	1q42.13	126518	1/27	1	0
FFC_CNVR_11	1	237152806	237190804	1q43	37998	1/27	1	0
FFC_CNVR_12	1	246837884	246852068	1q44	14184	2/27	2	0
FFC_CNVR_13	2	858450	1069320	2p25.3	210870	1/27	0	1
FFC_CNVR_14	2	1559257	1757565	2p25.3	198308	1/27	0	1
FFC_CNVR_15	2	4191253	4199818	2p25.3	8565	4/27	4	0
FFC_CNVR_16	2	9329183	9348229	2p25.1	19046	1/27	0	1

CNVR ID	Chr	CNVR Start pos	CNVR End pos	Chr band	CNVR length	CNV freq in cases	DEL freq in cases	DUPL freq in cases
FFC_CNVR_17	2	18035293	18056322	2p24.2	21029	1/27	0	1
FFC_CNVR_18	2	34556561	34580068	2p22.3	23507	3/27	2	1
FFC_CNVR_19	2	51780147	51781546	2p16.3	1399	3/27	3	0
FFC_CNVR_20	2	52613957	52637176	2p16.3	23219	4/27	4	0
FFC_CNVR_21	2	89166734	91029672	2p11.2-2p11.1	1862938	1/27	1	0
FFC_CNVR_22	2	141764229	141880892	2q22.1	116663	1/27	1	0
FFC_CNVR_23	2	146583025	146592386	2q22.3	9361	1/27	1	0
FFC_CNVR_24	2	180123158	180129913	2q31.2	6755	1/27	1	0
FFC_CNVR_25	2	195689483	195689951	2q32.3	468	1/27	1	0
FFC_CNVR_26	2	212065237	212115322	2q34	50085	1/27	1	0
FFC_CNVR_27	2	228491220	228497825	2q36.3	6605	1/27	1	0
FFC_CNVR_28	3	267992	1746198	3p26.3	1478206	1/27	0	1
FFC_CNVR_29	3	6626929	6629060	3p26.1	2131	1/27	1	0
FFC_CNVR_30	3	37954886	37957761	3p22.2	2875	1/27	1	0
FFC_CNVR_31	3	60316742	61638818	3p14.2	1322076	1/27	0	1
FFC_CNVR_32	3	65166887	65187636	3p14.1	20749	2/27	2	0
FFC_CNVR_33	3	71137852	71148637	3p14.1	10785	1/27	0	1
FFC_CNVR_34	3	75502426	75719139	3p12.3	216713	5/27	5	0
FFC_CNVR_35	3	89489946	89499861	3p11.1	9915	1/27	1	0
FFC_CNVR_36	3	90463777	90483521	3p11.1	19744	1/27	0	1
FFC_CNVR_37	3	113572982	113626716	3q13.2	53734	1/27	0	1
FFC_CNVR_38	3	163614849	163641981	3q26.1	27132	2/27	2	0
FFC_CNVR_39	3	164004033	164101579	3q26.1	97546	6/27	5	1
FFC_CNVR_40	3	166524485	166560107	3q26.1	35622	3/27	3	0
FFC_CNVR_41	3	174742050	174771975	3q26.31	29925	1/27	0	1
FFC_CNVR_42	3	176564661	176566093	3q26.31	1432	4/27	4	0
FFC_CNVR_43	3	191220845	191221392	3q28	547	5/27	5	0
FFC_CNVR_44	4	34472964	34499424	4p15.1	26460	1/27	1	0
FFC_CNVR_45	4	44663360	44699744	4p13	36384	1/27	0	1
FFC_CNVR_46	4	63352170	63357704	4q13.1	5534	3/27	3	0
FFC_CNVR_47	4	64380191	64389207	4q13.1	9016	1/27	1	0
FFC_CNVR_48	4	66269296	66311570	4q13.1-4q13.2	42274	1/27	0	1
FFC_CNVR_49	4	69097539	69179871	4q13.2	82332	5/27	4	1
FFC_CNVR_50	4	115398433	115407950	4q26	9517	6/27	4	2
FFC_CNVR_51	4	157183142	157186835	4q32.1	3693	1/27	1	0
FFC_CNVR_52	4	161271979	161291569	4q32.1	19590	1/27	1	0
FFC_CNVR_53	4	162172873	162223074	4q32.2	50201	1/27	0	1
FFC_CNVR_54	5	822832	873185	5p15.33	50353	2/27	2	0
FFC_CNVR_55	5	2094665	2111143	5p15.33	16478	1/27	1	0
FFC_CNVR_56	5	8756085	8797557	5p15.2	41472	5/27	5	0

CNVR ID	Chr	CNVR Start pos	CNVR End pos	Chr band	CNVR length	CNV freq in cases	DEL freq in cases	DUPL freq in cases
FFC_CNVR_57	5	16083905	16087232	5p15.1	3327	1/27	0	1
FFC_CNVR_58	5	21734892	21743780	5p14.3	8888	1/27	0	1
FFC_CNVR_59	5	27647188	27668522	5p14.1	21334	1/27	1	0
FFC_CNVR_60	5	34335990	34349167	5p13.3	13177	1/27	0	1
FFC_CNVR_61	5	97074222	97127572	5q15	53350	5/27	5	0
FFC_CNVR_62	5	97304658	97450879	5q21.1	146221	1/27	1	0
FFC_CNVR_63	5	151498251	151499003	5q33.1	752	1/27	1	0
FFC_CNVR_64	6	27742619	27765626	6p22.1	23007	1/27	0	1
FFC_CNVR_65	6	29959422	30030278	6p21.33	70856	5/27	4	1
FFC_CNVR_66	6	31388080	31408775	6p21.33	20695	3/27	3	0
FFC_CNVR_67	6	31444849	31449319	6p21.33	4470	1/27	1	0
FFC_CNVR_68	6	32558099	32664508	6p21.32	106409	8/27	6	2
FFC_CNVR_69	6	32720605	32731201	6p21.32	10596	1/27	0	1
FFC_CNVR_70	6	32746293	32752531	6p21.32	6238	1/27	1	0
FFC_CNVR_71	6	62021131	62519883	6q11.1	498752	1/27	1	0
FFC_CNVR_72	6	67061239	67104015	6q12	42776	3/27	3	0
FFC_CNVR_73	6	79029649	79090197	6q14.1	60548	4/27	2	2
FFC_CNVR_74	6	87243160	87293586	6q14.3	50426	1/27	0	1
FFC_CNVR_75	6	120242668	120275676	6q22.31	33008	1/27	1	0
FFC_CNVR_76	6	141120817	141406165	6q24.1	285348	1/27	0	1
FFC_CNVR_77	7	29207970	29501850	7p15.1	293880	1/27	0	1
FFC_CNVR_78	7	29657608	29740129	7p15.1	82521	1/27	0	1
FFC_CNVR_79	7	61681059	62336389	7q11.21	655330	5/27	4	1
FFC_CNVR_80	7	70025889	70047255	7q11.22	21366	1/27	0	1
FFC_CNVR_81	7	100755083	100914175	7q22.1	159092	1/27	0	1
FFC_CNVR_82	7	110432667	110565807	7q31.1	133140	1/27	0	1
FFC_CNVR_83	7	110822475	111126464	7q31.1	303989	2/27	2	0
FFC_CNVR_84	8	584761	588391	8p23.3	3630	9/27	9	0
FFC_CNVR_85	8	3610796	3642669	8p23.2	31873	1/27	1	0
FFC_CNVR_86	8	7356405	7918888	8p23.1	562483	2/27	2	0
FFC_CNVR_87	8	15821208	15830711	8p22	9503	1/27	1	0
FFC_CNVR_88	8	16306880	16317263	8p22	10383	2/27	2	0
FFC_CNVR_89	8	17795520	17796439	8p22	919	1/27	1	0
FFC_CNVR_90	8	39356825	39544185	8p11.23-8p11.22	187360	14/27	5	9
FFC_CNVR_91	8	40304029	40308706	8p11.21	4677	2/27	2	0
FFC_CNVR_92	8	47647579	47654762	8q11.1	7183	1/27	0	1
FFC_CNVR_93	8	83443991	83456427	8q21.13	12436	1/27	1	0
FFC_CNVR_94	8	107157602	107184658	8q23.1	27056	1/27	1	0
FFC_CNVR_95	9	8327939	8335923	9p24.1	7984	1/27	0	1
FFC_CNVR_96	9	11631257	11667252	9p23	35995	1/27	1	0

CNVR ID	Chr	CNVR Start pos	CNVR End pos	Chr band	CNVR length	CNV freq in cases	DEL freq in cases	DUPL freq in cases
FFC_CNVR_97	9	43570503	44729464	9p11.2	1158961	6/27	5	1
FFC_CNVR_98	9	66666918	67023910	9q12	356992	1/27	1	0
FFC_CNVR_99	9	81270069	81312437	9q21.31	42368	1/27	1	0
FFC_CNVR_100	9	100517321	100551961	9q22.33	34640	1/27	0	1
FFC_CNVR_101	9	113666416	113670063	9q31.3	3647	1/27	1	0
FFC_CNVR_102	9	119559699	119563999	9q33.1	4300	1/27	0	1
FFC_CNVR_103	10	35600595	35704462	10p11.21	103867	1/27	0	1
FFC_CNVR_104	10	47013328	47173619	10q11.22	160291	3/27	0	3
FFC_CNVR_105	10	47519753	47567295	10q11.22	47542	1/27	0	1
FFC_CNVR_106	10	51648436	51805221	10q11.23	156785	1/27	0	1
FFC_CNVR_107	10	56118892	56132477	10q21.1	13585	1/27	0	1
FFC_CNVR_108	10	66980652	66982114	10q21.3	1462	2/27	2	0
FFC_CNVR_109	10	96490096	96537453	10q23.33	47357	1/27	1	0
FFC_CNVR_110	10	135106317	135227438	10q26.3	121121	2/27	0	2
FFC_CNVR_111	11	31491931	31607765	11p13	115834	1/27	0	1
FFC_CNVR_112	11	39064163	39095687	11p12	31524	1/27	1	0
FFC_CNVR_113	11	49685221	49701670	11p11.12	16449	1/27	1	0
FFC_CNVR_114	11	50654023	51028075	11p11.12	374052	1/27	0	1
FFC_CNVR_115	11	54468566	55096955	11q11	628389	3/27	2	1
FFC_CNVR_116	11	55124465	55209499	11q11	85034	8/27	7	1
FFC_CNVR_117	11	55217364	55774484	11q11	557120	1/27	0	1
FFC_CNVR_118	11	85982050	85984049	11q14.2	1999	2/27	2	0
FFC_CNVR_119	11	100824741	100888177	11q22.1	63436	1/27	0	1
FFC_CNVR_120	11	103544199	104784378	11q22.3	1240179	1/27	0	1
FFC_CNVR_121	11	109964270	109980695	11q22.3	16425	1/27	1	0
FFC_CNVR_122	12	6114170	6159369	12p13.31	45199	1/27	1	0
FFC_CNVR_123	12	7891603	8005696	12p13.31	114093	2/27	1	1
FFC_CNVR_124	12	11372324	11500043	12p13.2	127719	1/27	1	0
FFC_CNVR_125	12	19384370	19455989	12p12.3	71619	1/27	0	1
FFC_CNVR_126	12	30129221	30132410	12p11.22	3189	1/27	1	0
FFC_CNVR_127	12	31157554	31298174	12p11.21	140620	2/27	0	2
FFC_CNVR_128	12	31901031	31954269	12p11.21	53238	3/27	0	3
FFC_CNVR_129	12	33193705	33197122	12p11.21	3417	3/27	3	0
FFC_CNVR_130	12	33717508	34692538	12p11.1	975030	1/27	0	1
FFC_CNVR_131	12	56756723	56785193	12q14.1	28470	1/27	1	0
FFC_CNVR_132	12	117436869	117495852	12q24.23	58983	1/27	1	0
FFC_CNVR_133	12	128374258	129046597	12q24.32 - 12q24.33	672339	1/27	0	1
FFC_CNVR_134	12	130296270	130390749	12q24.33	94479	1/27	1	0
FFC_CNVR_135	13	27410585	27412987	13q12.2	2402	1/27	0	1
FFC_CNVR_136	13	41984646	42008367	13q14.11	23721	1/27	0	1

CNVR ID	Chr	CNVR Start pos	CNVR End pos	Chr band	CNVR length	CNV freq in cases	DEL freq in cases	DUPL freq in cases
FFC_CNVR_137	13	56659471	56680301	13q21.1	20830	3/27	3	0
FFC_CNVR_138	13	68150996	68161739	13q21.33	10743	5/27	5	0
FFC_CNVR_139	13	84353807	84387072	13q31.1	33265	1/27	1	0
FFC_CNVR_140	13	88613376	88636971	13q31.2	23595	1/27	1	0
FFC_CNVR_141	13	94799350	94840021	13q32.1	40671	1/27	0	1
FFC_CNVR_142	14	18105752	18531500	14q11.1	425748	1/27	1	0
FFC_CNVR_143	14	19283777	19486339	14q11.2	202562	1/27	0	1
FFC_CNVR_144	14	36487191	36494521	14q13.3	7330	1/27	1	0
FFC_CNVR_145	14	40670768	40738084	14q21.1	67316	6/27	3	3
FFC_CNVR_146	15	18766242	20009688	15q11.2	1243446	5/27	4	1
FFC_CNVR_147	15	21601351	21612590	15q11.2	11239	2/27	2	0
FFC_CNVR_148	15	22070875	22219100	15q11.2	148225	1/27	0	1
FFC_CNVR_149	15	28294753	28524636	15q13.2	229883	1/27	1	0
FFC_CNVR_150	15	30532103	30620951	15q13.3	88848	1/27	1	0
FFC_CNVR_151	15	32518050	32595161	15q14	77111	2/27	2	0
FFC_CNVR_152	15	49921206	50062155	15q21.2	140949	1/27	0	1
FFC_CNVR_153	15	54579894	54582930	15q21.3	3036	1/27	1	0
FFC_CNVR_154	15	65721221	65747577	15q23	26356	1/27	1	0
FFC_CNVR_155	16	19858002	19862969	16p12.3	4967	4/27	4	0
FFC_CNVR_156	16	21432989	21746841	16p12.2-16p12.1	313852	1/27	1	0
FFC_CNVR_157	16	32818022	32848810	16p11.2	30788	1/27	1	0
FFC_CNVR_158	16	34344901	34464860	16p11.2-16p11.1	119959	1/27	0	1
FFC_CNVR_159	16	35043404	35126825	16p11.1	83421	3/27	3	0
FFC_CNVR_160	16	74114339	74131385	16q23.1	17046	1/27	0	1
FFC_CNVR_161	16	75280001	75401656	16q23.1	121655	1/27	1	0
FFC_CNVR_162	17	19456808	19475026	17p11.2	18218	1/27	1	0
FFC_CNVR_163	17	21304171	21369345	17p11.2	65174	1/27	0	1
FFC_CNVR_164	17	31462326	31490744	17q12	28418	1/27	0	1
FFC_CNVR_165	17	36928066	36944625	17q21.2	16559	1/27	1	0
FFC_CNVR_166	17	74233533	74345511	17q25.3	111978	1/27	0	1
FFC_CNVR_167	18	2846536	2936291	18p11.32-18p11.31	89755	1/27	1	0
FFC_CNVR_168	18	36514913	36519446	18q12.3	4533	3/27	3	0
FFC_CNVR_169	18	64895268	64906488	18q22.1-18q22.2	11220	1/27	1	0
FFC_CNVR_170	19	20404485	20507068	19p12	102583	2/27	2	0
FFC_CNVR_171	19	47997996	48130590	19q13.31	132594	1/27	1	0
FFC_CNVR_172	19	48205107	48430180	19q13.31	225073	1/27	1	0
FFC_CNVR_173	19	59941382	60069820	19q13.42	128438	2/27	1	1
FFC_CNVR_174	20	11799	17094	20p13	5295	1/27	0	1

CNVR ID	Chr	CNVR Start pos	CNVR End pos	Chr band	CNVR length	CNV freq in cases	DEL freq in cases	DUPL freq in cases
FFC_CNVR_175	20	12758367	12838206	20p12.1	79839	1/27	0	1
FFC_CNVR_176	20	40700356	40724350	20q12	23994	1/27	1	0
FFC_CNVR_177	20	52081775	52088118	20q13.2	6343	1/27	1	0
FFC_CNVR_178	21	9730102	9849404	21p11.2	119302	1/27	1	0
FFC_CNVR_179	21	13391465	13929416	21q11.2	537951	1/27	0	1
FFC_CNVR_180	21	19517280	19577289	21q21.1	60009	1/27	0	1
FFC_CNVR_181	21	43647907	43653486	21q22.3	5579	1/27	0	1
FFC_CNVR_182	22	23999569	24240667	22q11.23	241098	4/27	2	2
FFC_CNVR_183	22	30341225	30387964	22q12.2	46739	1/27	1	0
FFC_CNVR_184	X	18622368	18709806	Xp22.13	87438	1/27	0	1
FFC_CNVR_185	X	141482050	141499457	Xq27.2	17407	1/27	0	1
FFC_CNVR_186	X	154049748	154056000	Xq28	6252	1/27	1	0

Supp. Table S4. Prioritized CNVRs predicted based on SNP array and subjected to experimental copy number typing using TaqMan qPCR assays in the discovery sample set

SNP array estimation								TaqMan qPCR estimation			
					CNV carriers (n)[a]				CNV carriers (n)[b]		
Chr. band	Inner Start, End[c]	Length (kb)[d]	Type	Genes in the CNV region	Cases	Controls		CNVR confirmed[e]	Cases	Controls	Reported in DGV[f]
1p36.33	1220136, 1269723	49.59	Del	*SCNN1D*, *ACAP3*, *PUSL1*, *CPSF3L*, *GLTPD1*, *TAS1R3*	4	0		Yes	Criteria not met[g]		Yes
2p11.2	90094257, 90120151	25.90	Del/Dup[h]	*IGKV* region	5	1		Yes	5	2	Yes
4q25	108065738, 108073210	7.47	Del	Intergenic; 108 kb 5'of *DKK2*	4	0		Yes	4	1	Yes
5p13.3	32107084, 32159517	52.43[i]	Dup	*PDZD2*, *GOLPH3*	3	0		Yes	5	0	Yes
6p21.33	32006603, 32006896	0.29	Del/Dup[h]	*CYP21A2*	4	1		Yes	Criteria not met[g]		Yes
7p11.2	55866443, 55905896	39.45	Del/Dup[h]	*SEPT14*	2	0		Yes	2	0	Yes
8p22	16262509, 16272892	10.38	Del	Intergenic; 212 kb 5'of *MSR1*	5	2		Yes	Criteria not met[g]		Yes
12p13.31	6243909, 6247564	3.66	Del	Intergenic; 10 kb 5' of *VWF*	3	1		Yes	Criteria not met[g]		Yes
14q32.33	106028569, 106112716	84.15[i]	Del	*IGHE*	3	0		Yes	Criteria not met[g]		Yes

[a]Based on QuantiSNP analysis of genome-wide SNP genotyping data of 43 RM cases and 27 fertile female controls.
[b]TaqMan qPCR was performed in the identical discovery sample set as in the SNP genotyping and CNV calling analysis.
[c]Minimal CNVR range overlapping between all CNV carriers. Genomic coordinates along the chromosome are according to hg19.
[d]Size of the minimal range within the CNVR overlapping between all CNV carriers based on SNP genotyping and CNV calling data.
[e]CNV region confirmed as copy number variable by TaqMan qPCR.
[f]CNV region reported as copy number variable in the Database of Genomic Variants (DGV). Presence in the DGV was considered as confirmation of the prioritized CNVR as true CNV locus.
[g]Although confirmed as copy number variable by TaqMan qPCR, the CNVR was excluded from further study due to inconsistent copy number data likely due to complex genomic context of the rearrangement (Supp. Figure S6).

[h]CNV regions characterized by both deletion or duplication events in studied subjects.
[i]Two TaqMan qPCR assays were applied for the quantification of the two largest regions among the prioritized CNVRs.
CNV, copy number variant; RM, recurrent miscarriage; Del, deletion; Dup, duplication; *SCNN1D* (MIM# 601328), *sodium channel, non-voltage-gated 1, delta subunit; ACAP3, ArfGAP with coiled-coil, ankyrin repeat and PH domains 3*; *PUSL1, pseudouridylate synthase-like 1*; *CPSF3L* (MIM# 611354), *cleavage and polyadenylation specific factor 3-like*; *GLTPD1* (MIM# 615467), *glycolipid transfer protein domain containing 1*; *TAS1R3* (MIM# 605865), *taste receptor, type 1, member 3; IGKV* (MIM# 146980), *Immunoglobulin kappa variable cluster; DKK2* (MIM# 605415), *Dickkopf 2 homolog; PDZD2* (MIM# 610697*), PDZ domain containing 2; GOLPH3* (MIM# 612207), *Golgi phosphoprotein 3; CYP21A2* (MIM# 613815), *cytochrome P450, family 21, subfamily A, polypeptide 2; SEPT14* (MIM# 612140), *septin 14; MSR1* (MIM# 153622), *macrophage scavenger receptor 1; VWF* (MIM# 613160), *von Willebrand factor; IGHE* (MIM# 147180), *immunoglobulin heavy constant epsilon.*

Supp. Table S5. Pre-designed TaqMan Copy Number Assays (Applied Biosystems, Foster City, CA, USA) used in the study

Genomic localization	Targeted locus	TaqMan assay ID	Amount per reaction (µl)	
			Target assay	Reference assay[a]
1p36.33	*SCNN1D*	Hs01457469_cn	0.25	0.25
2p11.2	Intergenic	Hs03220290_cn	1.00	1.00
4q25	*DKK2*	Hs03235237_cn	1.00	0.50
5p13.3	*PDZD2*	Hs02781158_cn	1.00	0.50
	GOLPH3	Hs01071257_cn	0.25	0.25
7p11.2	*SEPT14*	Hs03632689_cn	1.00	1.00
8p22	Intergenic	Hs03272025_cn	0.50	1.00
12p13.31	Intergenic	Hs03294089_cn	0.25	0.25
14q32.33	*IGHE*	Hs03871600_cn	0.25	0.25
	IGHE	Hs04438403_cn	0.25	0.25

[a]TaqMan Copy Number Reference Assay RNAse P (Applied Biosystems) was used as a reference to the target copy number assays.

Supp. Table S6. Sequences of primers used in the study

Primer/Probe name	5’-3’ sequence	Product length
CYP21A2 (6p21.33) TaqMan qPCR primers and probe[a]		
TaqMan_CYP21A2_F	ACCTGTCCTTGGGAGACTAC	132 bp
TaqMan_CYP21A2_R	TTACCTCACAGAACTCCTGGGT	
TaqMan_CYP21A2_probe	FAM - TCTGGAAAGCCCACA - MGB	
5p13.3 CNV breakpoint mapping with EvaGreen qPCR		
EvaGr_Assay1_F	CCGCCTTCGTGGTTGGT	70 bp
EvaGr_Assay1_R	ACACGCTTTTTTGGGTTGTGT	
EvaGr_Assay2_F	CCCTGCCCTATCTCCTTTCC	63 bp
EvaGr_Assay2_R	TGGAGATGGACTTGCTGATGAT	
EvaGr_Assay3_F	CTGAACTACAAGCCAAATTCTATTGC	79 bp
EvaGr_Assay3_R	GCTTGTGCTTAGTTGAGGTTTAGAAA	
EvaGr_Assay4_F	TCTGATCCACACGCTGACATG	67 bp
EvaGr_Assay4_R	TGAGAGAATAACGAGCTGTAGGGTTT	
EvaGr_ALB_F	TGTTGCATGAGAAAACGCCA	151 bp
EvaGr_ALB_R	TCTGCATGGAAGGTGAATGTTT	
5p13.3 CNV breakpoint junction-spanning control PCR (BP-PCR)		
BP_PCR_F	CGTAATTCAAATGCTCCATACAAG	555 bp
BP_PCR_R	TAGAGGAAAGTCTTCTAGGACAGG	
5p13.3 CNV breakpoint junction sequencing		
BP_seq_F	CTGATGCCTCCAGGGTATTT	
BP_seq_R	ACAGCCTCTCACCGTCACTC	

[a]Previously published by Parajes et al., 2007.

Supp. Table S7. Prevalence of the 2p11.2, 4q11.2 and 5p13.3 CNVs in Estonian RM cases and fertile controls

			No of carriers		% of carriers		
Location	Genes in CNVR	CNV type	Patients	Controls	Patients	Controls	OR (CI 95%)
2p11.2	*IGKV*	deletion	15/119	8/90	12.6	8.9	1.48 (0.60-3.66)
		duplication	1/119	1/90	0.8	1.1	0.75 (0.05-12.23)
4q25[a]	*DKK2*	deletion	10/119	9/90	8.4	10.0	0.83 (0.32-2.13)
5p13.3	*PDZD2, GOLPH3*	duplication	9/119	1/90	7.6	1.1	7.28 (0.90-58.60)[b]

[a]Intergenic CNV, 108 kb upstream from the closest gene, *DKK2.*
[b]CNV region with the strongest effect subjected to genetic association testing.

Supp. Table S8. Sex-stratified association analysis of the *PDZD2:GOLPH3* CNV at 5p13.3 with recurrent miscarriage in Estonia and Denmark

Subjects in association testing				Association testing[a]		
Control group	No of subjects/ carriers (%)	Case group	No of subjects/ carriers (%)	OR	CI (95%)	*P*-value
Association testing 1: full Estonian RM case-control sample						
Fertile women	90/1 (1.1)	All	119/9 (7.6)	7.28	0.91-58.56	0.062
		Men	39/3 (7.7)	7.42	0.75-73.68	0.087
		Women	80/6 (7.5)	7.22	0.85-61.25	0.070
Association testing 2: Estonian RM cases versus EGCUT cohort						
EGCUT all	1000/9 (0.9)	All	119/9 (7.6)	9.01	3.50-23.17	**$5.1x10^{-6}$**
EGCUT men	504/4 (0.8)	Men	39/3 (7.7)	10.42	2.25-48.33	**0.003**
EGCUT women	496/5 (1.0)	Women	80/6 (7.5)	7.96	2.37-26.75	**$7.9x10^{-4}$**
Replication study: Danish RM case-control sample						
Fertile women	115/2 (1.7)	All	439/19 (4.3)	2.56	0.59-11.13	0.211
		Men	210/4 (1.9)	1.10	0.20-6.08	0.916
		Women	229/15 (6.6)	3.96	0.89-17.62	0.071
Meta-analysis: Estonian and Danish RM cases and fertile controls						
Fertile women	205/3 (1.5)	All	558/27 (4.8)	3.62	1.09-12.05	**0.036**
		Men	249/7 (2.8)	2.17	0.55-8.57	0.268
		Women	309/21 (6.8)	4.82	1.42-16.40	**0.012**

Association testing was performed with duplication carriers versus non-carriers as established based on TaqMan qPCR and confirmed by junction-spanning PCR for RM cases and fertile controls (Supp. Materials and Methods). For EGCUT samples, duplication carriership was estimated based on SNP genotyping and CNV calling data.

[a]Association testing was performed using logistic regression analysis in Estonia and Denmark separately. The results were subsequently combined in the meta-analysis using inverse-variance method under fixed-effects model. Results with *P*-value <0.05 were considered significant and are indicated in bold.

Supp. Table S9. Z-score based meta-analysis in the Estonian and Danish case-control sample

Case subgroup	z-score	*P*-value
All cases	2.12	**0.034**
Men	0.98	0.329
Women	2.53	**0.011**
Primary RM	2.73	**0.006**
Secondary RM	1.90	0.058

Meta-analysis was performed by combining the logistic regression *P*-values, effect directions and effective sample sizes using METAL software as described by Willer et al., 2010. Inclusion of effective sample size corrects for the asymmetric case/control ratio in the Estonian and Danish sample sets.
Results with p-value <0.05 were considered significant and are indicated in bold.
